# The Hsp90 Co-Chaperone Sgt1 Governs *Candida albicans* Morphogenesis and Drug Resistance

**DOI:** 10.1371/journal.pone.0044734

**Published:** 2012-09-06

**Authors:** Rebecca S. Shapiro, Aimee K. Zaas, Marisol Betancourt-Quiroz, John R. Perfect, Leah E. Cowen

**Affiliations:** 1 Department of Molecular Genetics, University of Toronto, Toronto, Ontario, Canada; 2 Department of Medicine, Duke University Medical Center, Durham, North Carolina, United States of America; 3 Department of Molecular Genetics and Microbiology, Duke University Medical Center, Durham, North Carolina, United States of America; Stony Brook University, United States of America

## Abstract

The molecular chaperone Hsp90 orchestrates regulatory circuitry governing fungal morphogenesis, biofilm development, drug resistance, and virulence. Hsp90 functions in concert with co-chaperones to regulate stability and activation of client proteins, many of which are signal transducers. Here, we characterize the first Hsp90 co-chaperone in the leading human fungal pathogen, *Candida albicans*. We demonstrate that Sgt1 physically interacts with Hsp90, and that it governs *C. albicans* morphogenesis and drug resistance. Genetic depletion of Sgt1 phenocopies depletion of Hsp90, inducing yeast to filament morphogenesis and invasive growth. Sgt1 governs these traits by bridging two morphogenetic regulators: Hsp90 and the adenylyl cyclase of the cAMP-PKA signaling cascade, Cyr1. Sgt1 physically interacts with Cyr1, and depletion of either Sgt1 or Hsp90 activates cAMP-PKA signaling, revealing the elusive link between Hsp90 and the PKA signaling cascade. Sgt1 also mediates tolerance and resistance to the two most widely deployed classes of antifungal drugs, azoles and echinocandins. Depletion of Sgt1 abrogates basal tolerance and acquired resistance to azoles, which target the cell membrane. Depletion of Sgt1 also abrogates tolerance and resistance to echinocandins, which target the cell wall, and renders echinocandins fungicidal. Though Sgt1 and Hsp90 have a conserved impact on drug resistance, the underlying mechanisms are distinct. Depletion of Hsp90 destabilizes the client protein calcineurin, thereby blocking crucial responses to drug-induced stress; in contrast, depletion of Sgt1 does not destabilize calcineurin, but blocks calcineurin activation in response to drug-induced stress. Sgt1 influences not only morphogenesis and drug resistance, but also virulence, as genetic depletion of *C. albicans* Sgt1 leads to reduced kidney fungal burden in a murine model of systemic infection. Thus, our characterization of the first Hsp90 co-chaperone in a fungal pathogen establishes *C. albicans* Sgt1 as a global regulator of morphogenesis and drug resistance, providing a new target for treatment of life-threatening fungal infections.

## Introduction

Fungal pathogens have emerged as a leading cause of human mortality, particularly among the growing population of immunocompromised individuals. The frequency of invasive fungal infections has increased by over 200% in recent years [1], with staggering economic and public health costs; treatment expenditures in the United States alone exceed $2.6 billion and mortality rates range from 30–90%, depending on the pathogen and patient population [2], [3]. The opportunistic pathogen *Candida albicans* reigns as the leading cause of fungal infection and the fourth most common cause of hospital-acquired infection, with mortality rates approaching 50% [4]. *C. albicans* thrives within the oral cavity and gastrointestinal tracts of healthy humans, and can cause superficial infections of oral and vaginal epithelial cells, as well as lethal systemic infections through bloodstream dissemination and organ invasion in immunocompromised individuals [5].

The pathogenic prowess of *C. albicans* is achieved through numerous virulence traits. Factors influencing virulence include the expression of surface adhesins that mediate epithelial cell adherence [6], the secretion of host cell damaging proteases [7], the capacity to produce drug-resistant biofilms [8], and the ability to transition between yeast and filaments [9]. Morphogenetic flexibility is often coupled with virulence, as *C. albicans* mutants locked as either yeast or filaments are typically attenuated in virulence [10], [11], [12], [13], [14], [15], though there are cases where these traits may be uncoupled [16]. Morphogenesis is critical for biofilm formation [17], [18], and genes that govern morphogenesis are frequently co-regulated with those encoding virulence factors such as proteases and adhesins [19], highlighting the importance of morphogenesis for *C. albicans* pathogenicity. The transition from yeast to filamentous growth is induced by diverse environmental cues including serum, nutrient limitation, carbon dioxide, temperature, and pH, and complex cellular signaling pathways govern response to these cues [9], [20]. Amongst these pathways, the cAMP-protein kinase A (PKA) pathway plays a key role in mediating the transition from yeast to filamentous growth [9]. The mechanism by which some morphogenetic cues are sensed by the cAMP-PKA cascade is known, such as binding of the adenylyl cyclase Cyr1 to peptidoglycan in serum [21], though the mechanism underpinning responses to other cues, such as temperature, remains enigmatic.

The treatment of invasive fungal infections, including those caused by *C. albicans*, is notoriously challenging. This can largely be attributed to the close evolutionary relationship between fungal pathogens and their human hosts [22], [23], and the resulting limited selection of clinically effective antifungal drugs. The most widely deployed class of antifungal drugs in clinical use is the azoles, which inhibit lanosterol 14α-demethylase, encoded by *ERG11* in *C. albicans*, thereby blocking the biosynthesis of ergosterol, the predominant sterol of fungal membranes [24], [25]. Azole-mediated inhibition of Erg11 further results in the accumulation of a toxic sterol intermediate, produced by Erg3, which disrupts membrane integrity and exerts a severe membrane stress on the cell [24], [25]. The widespread deployment and prophylactic use of azoles, coupled with their fungistatic activity, results in conditions that favour the evolution of antifungal drug resistance [24], [26]. Resistance mechanisms that minimize the impact of the drug on the cell include overexpression of drug efflux pumps, or alteration of the drug target, Erg11. Resistance mechanisms that minimize drug toxicity include alterations in the ergosterol biosynthesis pathway, including loss of function of Erg3, which blocks the accumulation of the toxic sterol [24], [25]. Mechanisms that reduce drug toxicity are frequently dependent on cellular stress responses that are crucial for tolerating the membrane stress exerted by azoles [24], [25]. The newest class of antifungal in clinical use is the echinocandins, which inhibit (1,3)-β-D-glucan synthase, thereby disrupting integrity of the fungal cell wall and resulting in cell wall stress [24], [25]. The most common mechanism of echinocandin resistance is mutation in *FKS1,* encoding the catalytic subunit of (1,3)-β-D-glucan synthase [27]. Cellular stress responses are required for Fks1-mediated resistance, as well as for basal tolerance to the cell wall damage exerted by the echinocandins [9], further illustrating the paradigm in which stress response circuitry enables crucial responses to antifungal drug exposure.

A key mediator of circuitry controlling nearly all facets of *C. albicans* pathobiology, including morphogenesis, biofilm formation, virulence and antifungal drug resistance is the molecular chaperone Hsp90 [9], [28]. Hsp90 is a highly conserved and essential chaperone that regulates the form and function of diverse client proteins in all eukaryotes [29], [30]. Many Hsp90 client proteins are key regulators of cellular signaling, including protein kinases and transcription factors [29], [31], rendering Hsp90 poised to govern diverse cellular processes. In *C. albicans*, inhibition of Hsp90 induces a temperature-dependent morphogenetic transition from yeast to filamentous growth via several cellular circuits, including the cell cycle regulation [32], the Pho85-Pcl1-Hms1 pathway [33], and the cAMP-PKA signaling cascade [34]. Though key components of PKA signaling are required for filamentous growth induced by Hsp90 depletion [34], the molecular mechanism by which Hsp90 represses cAMP-PKA signaling remains unknown. In addition to effects on morphogenesis, inhibiting Hsp90 function in *C. albicans* blocks the emergence and maintenance of resistance to azoles, and enhances the efficacy of azoles in vivo [35], [36]. Compromise of Hsp90 function also abrogates *C. albicans* echinocandin resistance, renders this otherwise fungistatic class of drug fungicidal, and improves echinocandin efficacy in a murine model of disseminated infection [37]. Under planktonic growth conditions, Hsp90 enables drug resistance by stabilizing the catalytic subunit of the protein phosphatase calcineurin, Cna1, as well as the terminal mitogen-activated protein kinase (MAPK) of the protein kinase C (PKC) cell wall integrity pathway, Mkc1 [37], [38]. Compromising Hsp90 function also abrogates azole resistance of *C. albicans* biofilms, though in a manner distinct from effects on Cna1 and Mkc1 stability [28]. Consistent with its central role in mediating these pathogenicity traits, depletion of *C. albicans* Hsp90 attenuates virulence in a murine model of systemic fungal infection [34].

Importantly, Hsp90 does not function autonomously, but rather interacts with a battery of co-chaperones that regulate its activity as well as its interaction with client proteins. Hsp90 co-chaperones can perform different functions, from regulating Hsp90′s ATPase activity to altering its localization and trafficking, and can have dramatic effects on Hsp90 client folding and function, though the mechanisms by which they do so remain largely enigmatic [39]. For instance, mammalian Hsp90 co-chaperones Aha1 and p23 differentially modulate Hsp90-dependent stability of the mutant cystic fibrosis transmembrane conductance regulator (CFTR) [40]. Many Hsp90 co-chaperones act as client adaptor proteins, by specifically recruiting select client proteins to Hsp90. For instance, recruitment of protein kinases to Hsp90 requires the co-chaperone Cdc37, which interacts simultaneously with the kinase and Hsp90, and modulates Hsp90′s ATPase activity [41], [42]. The co-chaperone Sgt1 also functions as an adaptor protein, and binds Hsp90 concurrent with binding a diverse set of client proteins, including Skp1, an essential component of the kinetochore complex [43], [44], as well as the adenylyl cyclase Cyr1 in the model yeast *Saccharomyces cerevisiae* [45]. Hsp90 co-chaperones such as Cdc37 and Sgt1 have been identified in diverse eukaryotes, including plants, animals and model fungi, but have yet to be characterized in *C. albicans*. Given that Hsp90 is a global regulator of *C. albicans* biology and disease, the next frontier is to identify co-chaperones modulating Hsp90 function in this fungal pathogen.

Here, we characterize the first Hsp90 co-chaperone in the fungal pathogen *C. albicans*. We establish that Sgt1 regulates *C. albicans* morphogenesis and drug resistance, providing a powerful new therapeutic target for the treatment of life-threatening fungal infections.

## Results

### Genetic depletion of Sgt1 induces filamentation, wrinkly colony morphology and invasive growth

**Figure 1:**
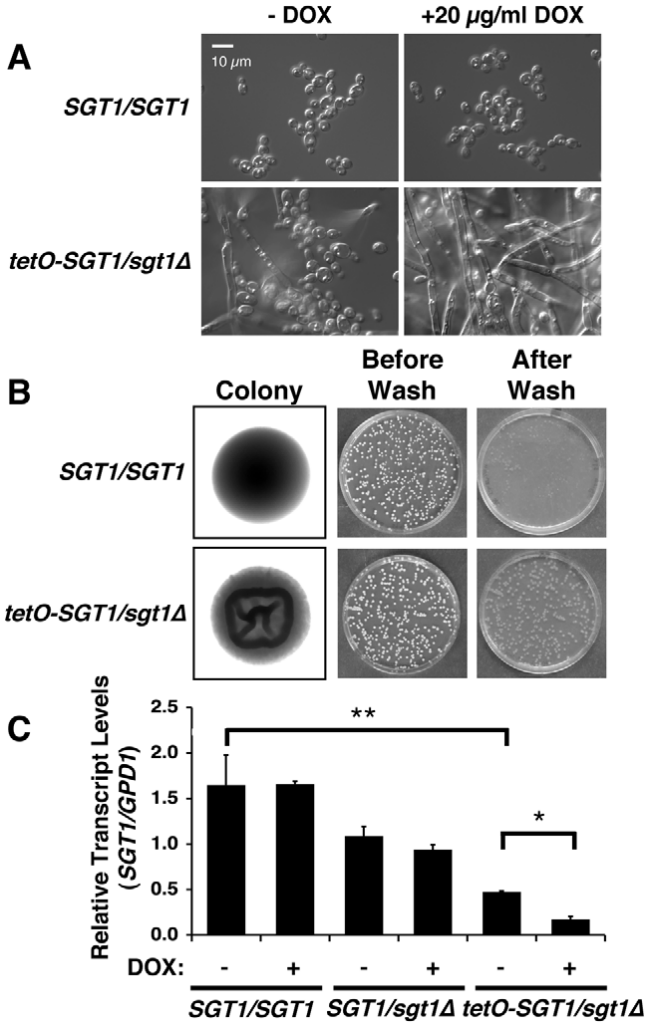
Genetic depletion of Sgt1 induces filamentation, wrinkly colony morphology and invasive growth. (A) Genetic depletion of Sgt1 induces filamentation. Sgt1 levels were reduced by growth overnight in 20 µg/ml doxycycline, followed by subculture in fresh medium with 20 µg/ml doxycycline. (B) Genetic depletion of Sgt1 in *the tetO-SGT1/sgt1*Δ strain leads to wrinkly colony morphology and invasive growth. Cells were plated on YPD agar and grown at 37°C for 3 days. Colonies were washed from the plates with H_2_O. (C) The *tetO-SGT1/sgt1*Δ strain has lower *SGT1* transcript levels compared to wild type in the absence of doxycycline (** indicates *P<*0.01, ANOVA), and *SGT1* levels are completely depleted upon treatment with doxycycline (* indicates *P<*0.05, ANOVA). Wild type, *SGT1/sgt11*Δ, or *tetO-SGT1/sgt1*Δ cells grown overnight in 20 µg/ml doxycycline, followed by subculture in fresh medium with 20 µg/ml doxycycline and grown until mid-log phase, as indicated. *SGT1* transcript levels were normalized to *GPD1*. Data are means ± standard deviations for triplicate samples.

As the mechanism by which Hsp90 modulates *C. albicans* morphogenesis via the cAMP-PKA cascade remained elusive, we assessed whether Hsp90 repressed PKA signaling via co-chaperones. Our previous work suggested that Hsp90 might interact with the protein kinase PKA or the adenylyl cyclase Cyr1 [34]. As a consequence, we focused on Cdc37 and Sgt1, co-chaperones that are well-established in other species to mediate Hsp90′s interaction with kinases and an adenylyl cyclase, respectively [45], [46]. As both *CDC37* and *SGT1* are essential in *S. cerevisiae* [44], [47], we created genetically repressible conditional mutants of *CDC37* and *SGT1* in *C. albicans*. For *CDC37* depletion, we engineered a strain in which the only allele of *CDC37* was regulated by the *MAL2* promoter, where transcription was repressed by glucose and induced by maltose (data not shown). For *SGT1* depletion, we engineered a strain in which the only allele of *SGT1* was regulated by the *tetO* promoter, where transcription was repressed by tetracycline or the analog doxycycline. Cdc37 depletion upon growth of the *MAL2p-CDC37/cdc37*Δ strain in the presence of glucose did not induce filamentation (Figure S1A), suggesting that it is not the link between Hsp90 and cAMP-PKA signaling. In contrast, doxycycline-mediated depletion of Sgt1 in the *tetO-SGT1/sgt1*Δ strain induced a transition from yeast to filamentous growth, in the absence of other inducing cues (Figure 1A and S1B). Notably, the filaments observed upon Sgt1 depletion bore a striking resemblance to those formed upon Hsp90 depletion [34]. Further, the *tetO-SGT1/sgt1*Δ strain produced wrinkly colonies on solid medium at 37°C (Figure 1B), as with depletion of Hsp90 [34]. The wrinkly colony morphology was coupled with invasion into agar, as *tetO-SGT1/sgt1*Δ colonies invaded agar and remained embedded in the plates despite washing, in contrast to the non-invasive colonies of the wild type (Figure 1B). That wrinkly colony morphology and invasive growth occurred in the absence of doxycycline is consistent with lower *SGT1* transcript levels in the *tetO-SGT1/sgt1*Δ strain even in the absence of doxycycline, consistent with the finding that expression of *SGT1* from the *tetO* promoter is lower than from the the native *SGT1* promoter (*P<*0.05, ANOVA, Bonferroni's Multiple Comparison Test, Figure 1C). Doxycycline-mediated transcriptional repression of *SGT1* led to reduced growth rates (Figure S2A), but had little effect on stationary phase cell density or viability under the conditions used in our assays (Figures S2B and S2C). Thus, genetic depletion of Sgt1, but not Cdc37, phenocopies depletion of Hsp90, and induces yeast to filament morphogenesis, wrinkly colony morphology, and invasive growth.

### Sgt1 interacts with Hsp90 in *C. albicans*

**Figure 2:**
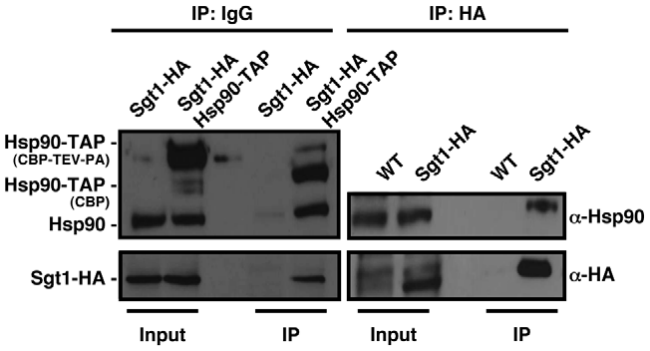
Sgt1 physically interacts with Hsp90 in *C. albicans*. Hsp90 and Sgt1 physically interact as measured by reciprocal co-immunoprecipitation of epitope-tagged proteins. Left panel, immunoprecipitation of Hsp90-TAP with anti-IgG agarose co-purified Sgt1-HA, while Sgt1-HA did not immunoprecipitate with anti-IgG agarose in control cells harbouring only untagged Hsp90. Right panel, immunoprecipitation of Sgt1-HA with anti-HA agarose co-purified Hsp90, while Hsp90 did not immunoprecipitate in control cells harbouring only untagged Sgt1.

The phenotypic similarities between genetic depletion of Hsp90 and Sgt1, suggest that Sgt1 may provide the link between Hsp90 and cAMP-PKA signaling. To determine if Sgt1 physically interacts with Hsp90 in *C. albicans*, as it does in species as diverse as *S. cerevisiae*, humans, and plants [43], [48], [49], we performed reciprocal co-immunoprecipitation between epitope-tagged versions of Hsp90 and Sgt1. We utilized an *HSP90* allele tagged at the C-terminus with a tandem affinity purification (TAP) tag [37], consisting of a calmodulin binding peptide (CBP), a tobacco etch virus (TEV) protease cleavage site, and Protein A, which binds IgG; the *SGT1* allele was tagged at the C-terminus with a haemagglutinin (HA) tag. Immunoprecipitation with anti-IgG agarose, followed by TEV protease cleavage, co-purified TAP-tagged Hsp90, as well as HA-tagged Sgt1; for the control strain lacking the tagged *HSP90* allele, Sgt1-HA was present in the input, but was not immunoprecipitated (Figure 2, left panel). To further validate this interaction, we performed the reciprocal co-immunoprecipitation using HA-tagged Sgt1 and untagged Hsp90. Immunoprecipitation with anti-HA agarose co-purified HA-tagged Sgt1, as well as wild-type Hsp90; for the control strain lacking the tagged *SGT1* allele, Hsp90 was present in the input, but was not immunoprecipitated (Figure 2, right panel). Thus, reciprocal co-immunoprecipitation reveals a physical interaction between Hsp90 and Sgt1, and identifies the first putative Hsp90 co-chaperone in *C. albicans.*

### Depletion of Sgt1 activates cAMP-PKA signaling, and Sgt1 interacts with the adenylyl cyclase Cyr1

**Figure 3:**
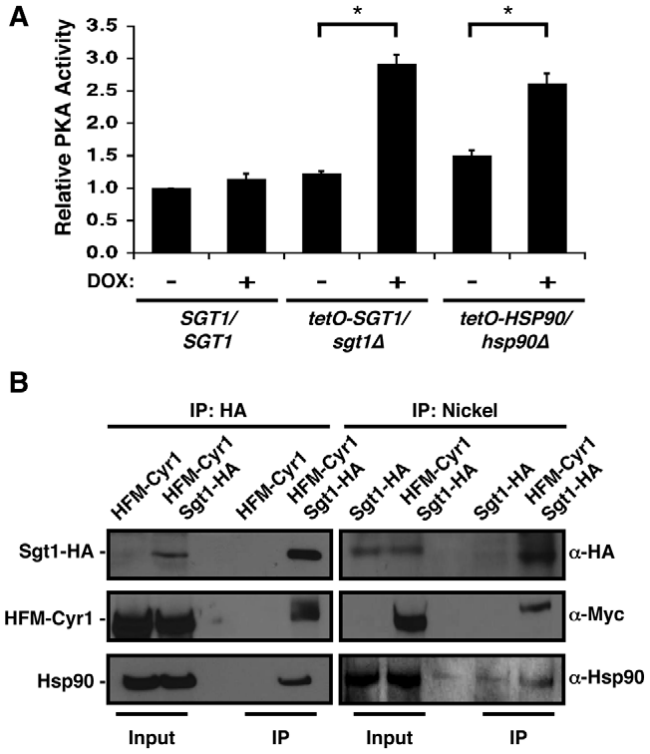
Depletion of Sgt1 activates cAMP-PKA signaling, and Sgt1 interacts with the adenylyl cyclase Cyr1. (A) Depletion of Sgt1 activates cAMP-PKA signaling. PKA activity was measured for cell extracts derived from the indicated strains. Sgt1 or Hsp90 levels were reduced by growth overnight in 20 µg/ml doxycycline, followed by subculture in fresh medium with 20 µg/ml doxycycline and growth until mid-log phase. Data are normalized against the level of PKA activity of wild type cell extracts, and are displayed as means ± standard deviations for triplicate samples. * Indicates *P<*0.01, ANOVA. (B) Sgt1 and Cyr1 physically interact as measured by reciprocal co-immunoprecipitation of epitope-tagged proteins. Left panel, immunoprecipitation of Sgt1-HA with anti-HA agarose co-purified 6x-His-FLAG-Myc (HFM)-Cyr1, while HFM-Cyr1 did not immunoprecipitate with anti-HA agarose in control cells harbouring only untagged Sgt1. Hsp90 also co-purified with Sgt1-HA, and was not purified in the controls with only untagged Sgt1. Right panel, immunoprecipitation of HFM-Cyr1 with anti-Nickel agarose co-purified Sgt1, while Sgt1 did not immunoprecipitate in control cells harbouring untagged Cyr1. Hsp90 was enriched in the immunoprecipitation of HFM-Cyr1 relative to the control immunoprecipiation with only untagged Cyr1.

Since depletion of Hsp90 induces filamentous growth by activating cAMP-PKA signaling [34], we assessed whether depletion of Sgt1 might also stimulate this pathway to induce filamentous growth. To determine whether PKA signaling was activated, we performed a PKA activity assay, which has been successfully used to monitor PKA signaling in *C. albicans* [50]. We used whole-cell protein extracts derived from wild-type, *tetO-SGT1/sgt1*Δ and *tetO-HSP90/hsp90*Δ cells grown in the presence or absence of doxycycline, to deplete Sgt1 or Hsp90. In this assay, a fluorescently labeled peptide that is a highly specific substrate for PKA is phosphorylated, causing a change in charge. The peptide is then separated into phosphorylated and non-phosphorylated forms by electrophoresis, and quantified by spectrophotometry. We found that genetic depletion of Sgt1, or depletion of Hsp90, both led to significantly increased PKA activity, relative to the wild type (*P<*0.01, ANOVA, Bonferroni's Multiple Comparison Test, Figure 3A). Therefore, depletion of Sgt1 phenocopies depletion of Hsp90, leading to activation of PKA signaling.

To determine the mechanism by which Hsp90 and Sgt1 modulate PKA signaling, we investigated whether Sgt1 physically interacted with the adenylyl cyclase Cyr1, as these two proteins are known to interact in *S. cerevisiae* [45]. To determine whether Sgt1 interacts with Cyr1 in *C. albicans*, we performed reciprocal co-immunoprecipitation between epitope-tagged Cyr1 and Sgt1. We utilized an allele of *CYR1*, N-terminally tagged with 6X-His-FLAG-Myc (HFM) [51], and the HA-tagged Sgt1 strain described above. Immunoprecipitation with anti-HA agarose co-purified HA-tagged Sgt1, HFM-tagged Cyr1, as well as untagged Hsp90 (Figure 3B, left panel). For the strain lacking the tagged *SGT1* allele, HFM-Cyr1 and Hsp90 were present in the input, but were not immunoprecipitated (Figure 3B, left panel). To further validate this interaction, we performed the reciprocal co-immunoprecipitation. Immunoprecipitation with anti-Nickel (Ni-NTA) agarose co-purified HFM-tagged Cyr1, as well as HA-tagged Sgt1, and untagged Hsp90. For the control strain lacking the tagged *CYR1* allele, Sgt1-HA was not immunoprecipitated; some Hsp90 was immunoprecipiated in this strain, though to a lesser extent than in the strain harboring HFM-Cyr1 (Figure 3B, right panel). Thus, reciprocal co-immunoprecipitation reveals a physical interaction between Sgt1, Cyr1 and Hsp90 in *C. albicans*, suggesting a mechanism by which Hsp90 and Sgt1 interact with Cyr1 in order to modulate PKA signaling, and morphogenesis.

### Sgt1 enables basal tolerance and *erg3*-mediated resistance to azoles

**Figure 4:**
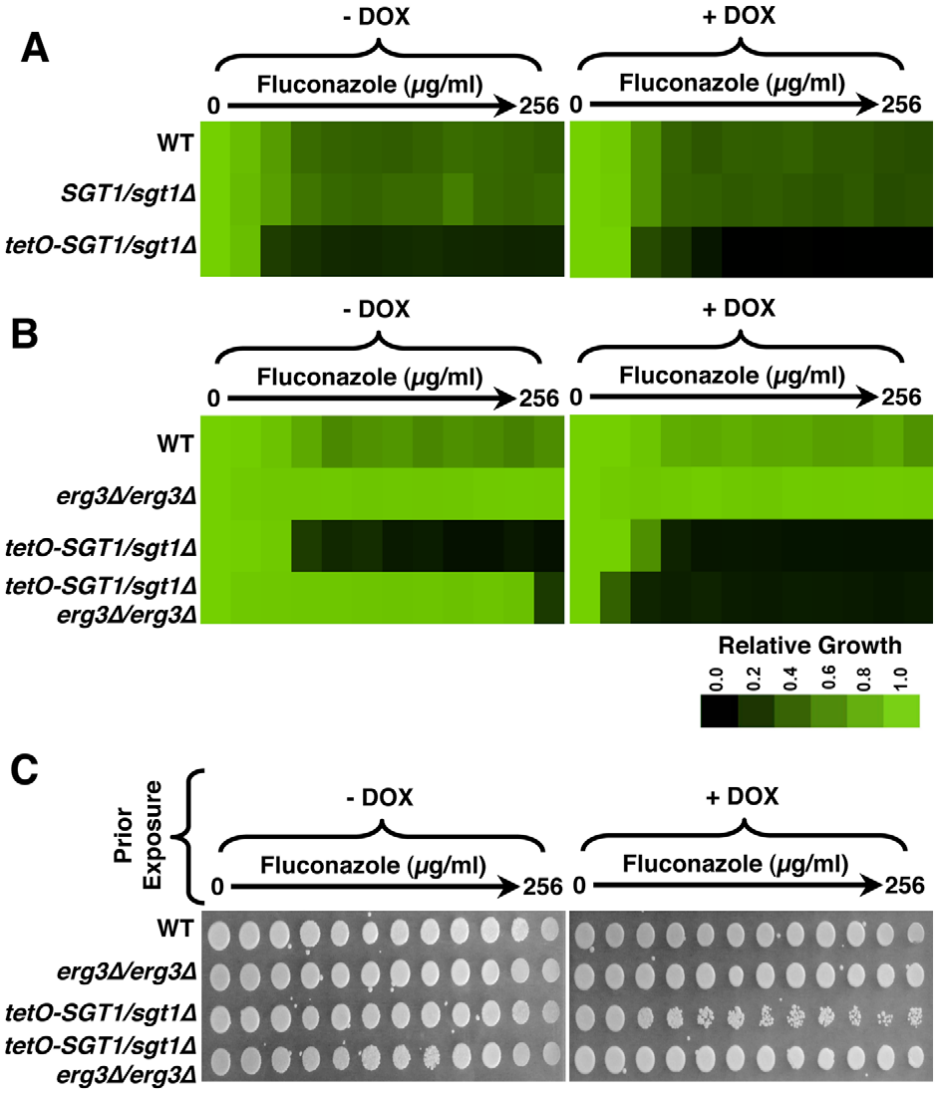
Sgt1 enables basal tolerance and *erg3*-mediated resistance to the azoles. (A) Reduced levels of Sgt1 renders cells sensitive to fluconazole in minimum inhibitory concentration (MIC) assays. Assays were performed in YPD medium with a gradient of fluconazole from 0 to 256 µg/ml, in two-fold dilutions, with or without a fixed concentration of 20 µg/ml doxycycline (DOX), as indicated. Growth was measured by absorbance at 600 nm after 48 hours at 30°C. Optical densities were averaged for duplicate measurements and normalized relative to the no fluconazole control. Data was quantitatively displayed with colour using Treeview (see colour bar). (B) Reduced levels of Sgt1 renders azole-resistant *erg3* mutants sensitive to fluconazole in MIC assays. Assays were performed in YPD medium with a gradient of fluconazole from 0 to 256 µg/ml, in two-fold dilutions, with a fixed concentration of 20 µg/ml doxycycline, as indicated. Growth was measured after 72 hours at 30°C. Data was analyzed as in part A. (C) Cells with reduced levels of Sgt1 remain viable after exposure to fluconazole. MIC assays were performed in YPD medium with a gradient of fluconazole from 0 to 256 µg/ml, in two-fold dilutions, with or without a fixed concentration of 20 µg/ml doxycycline, as indicated. Assays were grown for 48 hours at 30°C, and cells from the MIC assays were spotted onto YPD medium and incubated at 30°C for 48 hours before plates were photographed.

Having determined that depletion of Sgt1 phenocopies depletion of Hsp90 in terms of morphogenesis, we next assessed whether Sgt1 played a role in antifungal tolerance and resistance, as Hsp90 enables crucial cellular responses to drug-induced stress [35], [37]. To assess the impact of depletion of Sgt1 on tolerance to the most widely deployed class of antifungals, the azoles, we assayed growth of a wild-type strain, a strain heterozygous for *SGT1* (*SGT1/sgt1*Δ), as well as the *SGT1* doxycycline-repressible strain (*tetO-SGT1/sgt1*Δ) over a gradient of concentrations of the azole fluconazole relative to a drug-free control. The wild-type strain and *SGT1/sgt1*Δ mutant showed robust tolerance to all concentrations of fluconazole tested, while the *tetO-SGT1/sgt1*Δ strain showed increased susceptibility to fluconazole, even in the absence of doxycycline (Figure 4A). Depletion of Sgt1 with doxycycline had little additional effect (Figure 4A). This demonstrates that as with depletion of Hsp90 [35], depletion of Sgt1 increases susceptibility to azoles.

Next, we assessed if Sgt1 was also involved in antifungal resistance conferred by deletion of *ERG3*, which is known to cause robust resistance to azoles [52]. To determine the impact of depletion of Sgt1 on *erg3*-mediated azole resistance, we monitored growth of a wild-type strain and *erg3*Δ*/erg3*Δ mutant [53], as well as the *tetO-SGT1/sgt1*Δ strain and the *tetO-SGT1/sgt1*Δ strain with both alleles of *ERG3* deleted (*tetO-SGT1/sgt1*Δ *erg3*Δ*/erg3*Δ), over a gradient of concentrations of fluconazole. Consistent with our previous findings, the wild-type strain was tolerant to all concentrations of fluconazole tested, while the *tetO-SGT1/sgt1*Δ strain had increased susceptibility; doxycycline had little impact (Figure 4B). The *erg3*Δ*/erg3*Δ and *tetO-SGT1/sgt1*Δ *erg3*Δ*/erg3*Δ mutants were both robustly resistant to fluconazole; doxycycline-mediated depletion of Sgt1 rendered the *tetO-SGT1/sgt1*Δ *erg3*Δ*/erg3*Δ strain sensitive to fluconazole, while doxycycline had no effect on the control *erg3*Δ*/erg3*Δ strain (Figure 4B). Thus, Sgt1 is required for *erg3*-mediated azole resistance.

Inhibition of Hsp90 function transforms fluconazole from fungistatic to fungicidal [36]. To determine if the same was true for depletion of Sgt1, we exposed cells to a gradient of concentrations of fluconazole, in the presence of absence of a constant concentration of doxycycline, and then spotted cells on rich medium without any inhibitors. All strains tested were able to grow on rich medium following exposure to all concentrations of fluconazole tested, although doxycycline-mediated depletion of *SGT1* in the *tetO-SGT1/sgt1*Δ strain did reduce recovery following exposure to fluconazole (Figure 4C). Thus, depletion of Sgt1 has only a minor impact on the fungistatic activity of azoles, despite the central role for Sgt1 in basal tolerance and resistance to azoles.

### Depletion of Sgt1 enhances susceptibility to echinocandins, and transforms them from fungistatic to fungicidal

**Figure 5:**
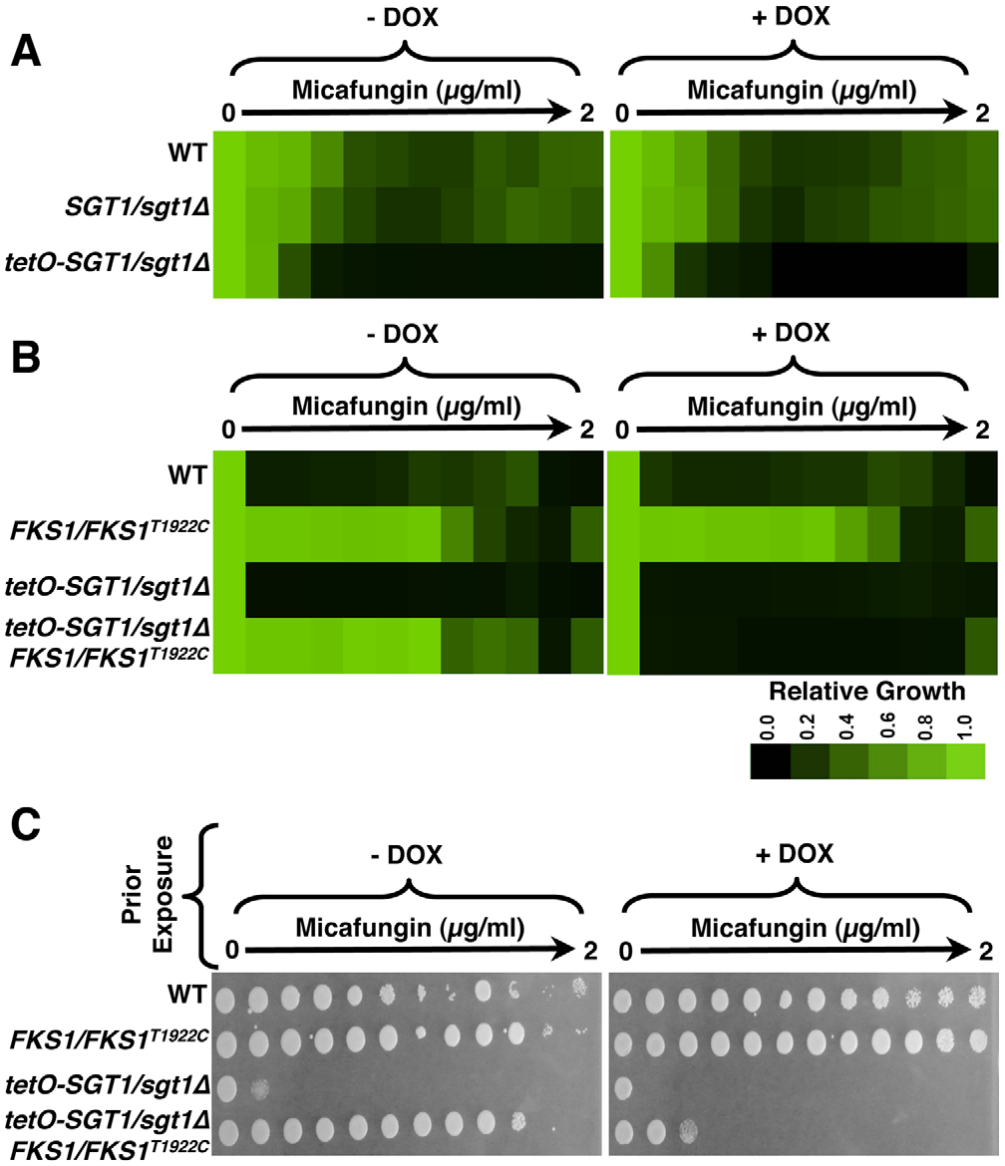
Reduction of Sgt1 levels enhances susceptibility to echinocandins and creates a fungicidal combination. (A) Reduced levels of Sgt1 enhances susceptibility to micafungin in minimum inhibitory concentration (MIC) assays. Assays were performed in YPD medium with a gradient of fluconazole from 0 to 2 µg/ml, in two-fold dilutions, with or without a fixed concentration of 20 µg/ml doxycycline (DOX), as indicated. Growth was measured after 72 hours at 30°C. Data was analyzed as in Figure 4. (B) Reduced levels of Sgt1 renders echinocandin-resistant *FKS1* mutants susceptible to micafungin in MIC assays. Assays were performed in YPD medium with a gradient of fluconazole from 0 to 8 µg/ml, in two-fold dilutions, with or without a fixed concentration of 20 µg/ml doxycycline, as indicated. Growth was measured after 72 hours at 30°C. Data was analyzed as in Figure 4. (C) Reduction of Sgt1 levels creates a fungicidal combination with micafungin. MIC assays were performed in YPD medium with a gradient of fluconazole from 0 to 2 µg/ml, in two-fold dilutions, with or without a fixed concentration of 20 µg/ml doxycycline, as indicated. Assays were grown for 72 hours at 30°C, and cells from the MIC assays were spotted onto YPD medium and incubated at 30°C for 48 hours before plates were photographed.

To determine if Sgt1 affects tolerance and resistance to the newest class of antifungal drug, the echinocandins, we assayed growth of a wild-type strain, a strain heterozygous for *SGT1* (*SGT1/sgt1*Δ), as well as the *SGT1* doxycycline-repressible strain (*tetO-SGT1/sgt1*Δ) over a gradient of concentrations of the echinocandin micafungin. The wild-type strain and *SGT1/sgt1*Δ mutant displayed tolerance to the gradient of micafungin, while the *tetO-SGT1/sgt1*Δ strain showed increased susceptibility to micafungin, even in the absence of doxycycline (Figure 5A), similar to what was observed with the azoles. Doxycycline-mediated depletion of Sgt1 had little further effect on susceptibility of the *tetO-SGT1/sgt1*Δ strain (Figure 5A). Therefore, reduced expression of Sgt1 increases echinocandin susceptibility.

Next, we determined if Sgt1 enabled echinocandin resistance conferred by mutation of *FKS1*, which is the most prevalent mechanism of echinocandin resistance [27], [54]. We utilized a well-characterized *FKS1* mutation (T1922C), shown to confer resistance to the echinocandins [55]. To assess the impact of depletion of Sgt1 on *FKS1*-mediated resistance to the echinocandins, we assayed growth of a wild-type strain and an *FKS1/FKS1^T1922C^* echinocandin-resistant strain, as well as the *tetO-SGT1/sgt1*Δ strain and the *tetO-SGT1/sgt1*Δ strain containing the *FKS1^T1922C^* mutation (*tetO-SGT1/sgt1*Δ *FKS1/FKS1^T1922C^*), over a gradient of concentrations of micafungin. The *FKS1/FKS1^T1922C^* mutant was resistant to micafungin relative to the wild-type strain (Figure 5B). The *tetO-SGT1/sgt1*Δ strain had increased susceptibility to micafungin relative to the wild type, while the *tetO-SGT1/sgt1*Δ *FKS1/FKS1^T1922C^* had resistance comparable to the *FKS1/FKS1^T1922C^* mutant (Figure 5B). Doxycycline-mediated depletion of Sgt1 in the *tetO-SGT1/sgt1*Δ *FKS1/FKS1^T1922C^* strain abrogated echinocandin resistance (Figure 5B), implicating Sgt1 as a key regulator of *FKS1*-mediated resistance to the echinocandins.

We next assessed if depletion of Sgt1 creates a fungicidal combination with echinocandins. Though the echinocandins are generally considered fungicidal against *C. albicans* [27], *C. albicans* can grow vigorously at intermediate echinocandin concentrations under laboratory conditions, and inhibition of Hsp90 transforms echinocandins from fungistatic to fungicidal [37]. We exposed cells to a gradient of concentrations of micafungin, in the presence or absence of a constant concentration of doxycycline, and then spotted cells onto rich medium without any inhibitors. The wild-type strain and *FKS1/FKS1^T1922C^* mutant were able to grow on rich medium following exposure to all concentrations of micafungin, in the presence or absence of doxycycline (Figure 5C). Genetic compromise of Sgt1 in the *tetO-SGT1/sgt1*Δ and micafungin-resistant *tetO-SGT1/sgt1*Δ *FKS1/FKS1^T1922C^* strains was cidal in combination with almost any dose of micafungin tested, even in the absence of doxycycline (Figure 5C). Therefore, genetic reduction of Sgt1 increases echinocandin susceptibility, and creates a fungicidal combination.

### The Hsp90 client protein calcineurin is dependent on Sgt1 for activation, but not stabilization

**Figure 6:**
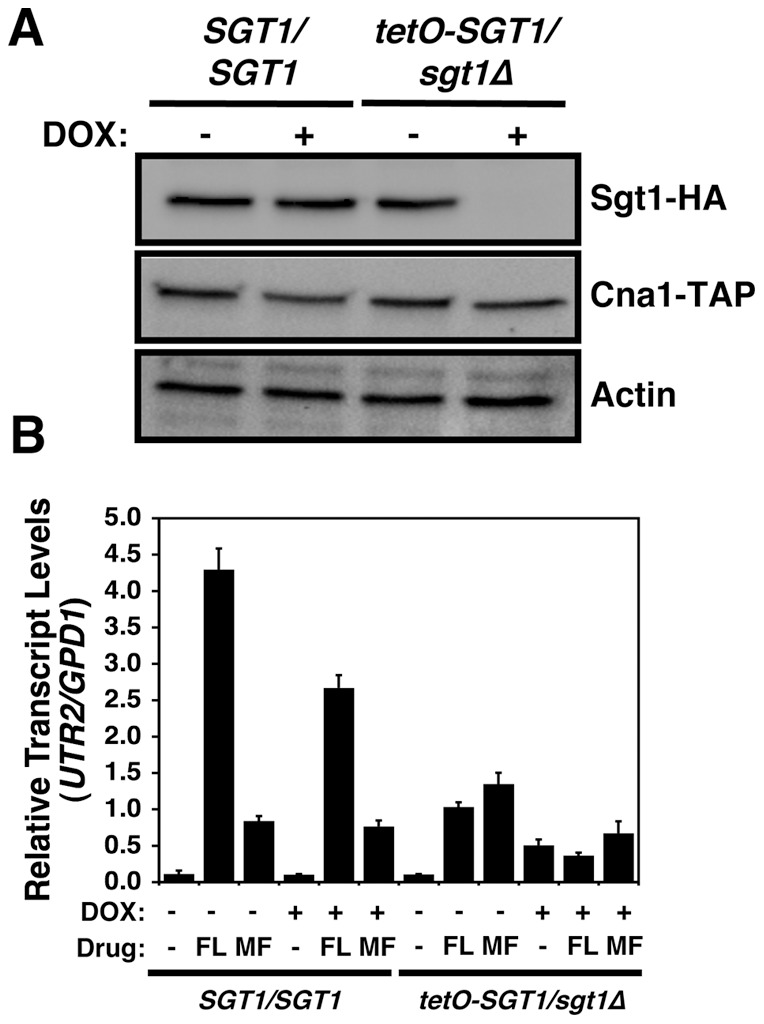
Hsp90 client protein Cna1 retains stability upon depletion of Sgt1, though Sgt1 is required for Cna1 activation. (A) Cna1 retains stability upon depletion of Sgt1. Sgt1 levels were reduced by growth overnight in 20 µg/ml doxycycline, followed by subculture in fresh medium with 20 µg/ml doxycycline and growth until mid-log phase. First panel, immune blot analysis of Sgt1 levels (50 µg protein loaded per well); and second panel, immune blot analysis of Cna1-TAP levels (50 µg protein loaded per well). Actin was used as a loading control. (B) Sgt1 is required for Cna1 activation in response to fluconazole or micafungin. Transcript levels of a Cna1-dependent gene, *UTR2*, were measured for the wild-type (*SGT1/SGT1*) or *tetO-SGT1/sgt1*Δ strains by quantitative RT-PCR after growth in rich medium at 30°C with or without 20 µg/ml doxycycline (DOX), 16 µg/ml fluconazole (FL), and 30 ng/ml micafungin (MF), as indicated. *UTR2* transcript levels were normalized to *GPD1*. Data are means ± standard deviations for triplicate samples.

To assess the mechanism by which depletion Sgt1 influences antifungal drug resistance, we focused on a key Hsp90 client protein implicated in drug resistance of *C. albicans*, calcineurin. The catalytic subunit of the protein phosphatase calcineurin, Cna1, depends on Hsp90 for its stability (Figure S3A, [37]). To assess whether Sgt1 was also required for Cna1 stability, we monitored protein levels of TAP-tagged Cna1 in a wild-type strain, the *tetO-SGT1/sgt1*Δ strain, as well as the *MAL2-SGT1/sgt1*Δ strain under conditions where Sgt1 is depleted. In the absence of Sgt1 depletion, all strains had comparable levels of Sgt1 and Cna1 (Figure 6A and Figure S3B). Genetic depletion of Hsp90 leads to a corresponding depletion of Cna1 (Figure S3A, [37]). However, depletion of Sgt1 had no impact on levels of Cna1 (Figure 6A and Figure S3B), indicating that Sgt1 is not required for Cna1 stability, in contrast to Cna1 dependence on Hsp90.

To determine if Sgt1 was required for activation, rather than stability of Cna1, we monitored transcript levels of a Cna1-dependent gene, *UTR2* [56], which is known to be upregulated upon Cna1 activation by azoles or echinocandins [37], [38]. In a wild-type strain, treatment with fluconazole or micafungin activated Cna1 (*P<*0.01, ANOVA, Bonferroni's Multiple Comparison Test), as measured by an increase in *UTR2* transcript levels (Figure 6B). While doxycycline caused a modest reduction of *UTR2* induction in response to fluconazole in the wild-type strain (*P*<0.001, ANOVA), doxycycline-mediated depletion of Sgt1 in the *tetO-SGT1/sgt1*Δ strain blocked calcineurin activation in response to fluconazole (*P*<0.01, ANOVA, Figure 6B). Doxycycline had no impact on *UTR2* induction in response to micafungin, but doxycycline-mediated depletion of Sgt1 in the *tetO-SGT1/sgt1*Δ strain blocked calcineurin activation in response to micafungin (*P*<0.01, ANOVA, Figure 6B). Thus, Sgt1 does not affect stability of the Hsp90 client protein calcineurin, but is required for calcineurin activation in response to drug-induced stress, providing a mechanism by which Sgt1 regulates drug resistance.

### Genetic depletion of *C. albicans* Sgt1 leads to reduced fungal burden in a murine model of disseminated infection

**Figure 7:**
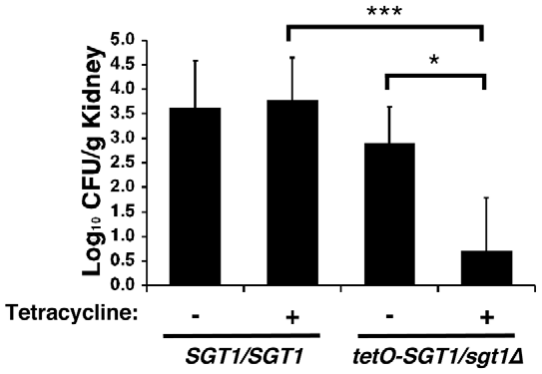
Genetic depletion of Sgt1 leads to reduced kidney fungal burden in a murine model of disseminated candidiasis. Genetic depletion of Sgt1 leads to reduced kidney fungal burden in a murine model of disseminated infection. CD1 mice were infected with 2.1×10^6^ CFU/ml of the wild-type strain or *tetO-SGT1/sgt1*Δ strain and were treated with tetracycline, as indicated. * Indicates *P<*0.05, *** indicates *P<*0.0001, Kruskal-Wallis test.

Sgt1 is a promising therapeutic target given its importance for morphogenesis and drug resistance. To assess the impact of depletion of Sgt1 on virulence, we employed a murine model of systemic infection, in which inoculum of *C. albicans* is delivered via tail vein injection, and progresses from the bloodstream to deep-seated infection in organs such as the kidney [34], [36]. Mice were infected with the wild-type strain or the *tetO-SGT1/sgt1*Δ strain, with or without tetracycline in the drinking water, and fungal burden in the mouse kidneys was monitored three days after infection. Tetracycline had no impact on kidney fungal burden of mice infected with the wild-type strain, but significantly reduced fungal burden of mice infected with the *tetO-SGT1/sgt1*Δ strain (Figure 7, *P<*0.05, Kruskal-Wallis test). Similarly, mice treated with tetracycline had significantly reduced kidney fungal burden when infected with *tetO-SGT1/sgt1*Δ, compared to those infected with the wild type (Figure 7, *P<*0.0001, Kruskal-Wallis test). Thus, depletion of Sgt1 leads to reduced kidney fungal burden, implicating Sgt1 as a potential therapeutic target for the treatment of *C. albicans* infections.

## Discussion

Our results identify Sgt1, the first Hsp90 co-chaperone characterized in *C. albicans*, as a global regulator of circuitry controlling morphogenesis and drug resistance, with broad therapeutic potential. Genetic depletion of Sgt1 phenocopies depletion of Hsp90, inducing a yeast to filament transition, wrinkly colony morphology and invasive growth (Figure 1). Sgt1 likely controls these morphogenetic traits by bridging two important regulators of morphogenesis: Hsp90 and the cAMP-PKA signaling cascade. Sgt1 physically interacts with both Hsp90 and the adenylyl cyclase Cyr1 (Figure 2 and Figure 3), and depletion of Sgt1 phenocopies depletion of Hsp90, activating PKA signaling (Figure 3), thus providing a molecular mechanism by which Hsp90 orchestrates morphogenesis via PKA signaling. Sgt1 is also a key regulator of Hsp90-dependent resistance to the two most widely deployed classes of antifungals, the azoles and the echinocandins (Figure 4 and Figure 5). Sgt1 enables both tolerance and *erg3*-mediated resistance to azoles (Figure 4). Depletion of Sgt1 abrogates basal tolerance and *FKS1^T1922C^*-mediated resistance to echinocandins, and transforms echinocandins from fungistatic to fungicidal (Figure 5). Though Sgt1 and Hsp90 share a conserved impact on drug resistance phenotypes, the mechanism involved are distinct. Depletion of Hsp90 leads to destabilization of a key client protein implicated in drug resistance, calcineurin [37], while depletion of Sgt1 does not alter calcineurin stability (Figure 6). Sgt1 is required for activation of calcineurin in response to drug-induced stress (Figure 6), suggesting a mechanism by which Sgt1 regulates antifungal drug resistance. Similar to Hsp90, Sgt1 also plays an important role in fitness in a murine model of infection (Figure 7), thus establishing this co-chaperone as a promising target for antifungal therapy.

Together, this work implicates Sgt1 as a central regulator of the Hsp90 chaperone machine. Importantly, though Hsp90 co-chaperones remain largely uncharacterized in *C. albicans*, it is apparent that not all Hsp90 co-chaperones influence these Hsp90-dependent traits, as depletion of the co-chaperone Cdc37 does not impact on *C. albicans* morphogenesis (Figure S1). Notably, Hsp90 does not regulate *C. albicans* morphogenesis and antifungal drug resistance via a single pathway, but rather has pleiotropic effects, consistent with its function as a central hub of protein homeostasis and regulatory circuitry. In *S. cerevisiae*, Hsp90 interacts with ∼10% of the proteome [57], and the chaperone network is equally complex in *C. albicans* [58]. Elucidating the impact of Sgt1 and other co-chaperones on Hsp90 targets on a more global scale will reveal fundamental insight into the mechanisms modulating function of the Hsp90 chaperone machine.

Through its interactions with both Cyr1 and Hsp90, *C. albicans* Sgt1 couples temperature sensing with morphogenesis. Hsp90, Sgt1, and Cyr1 may interact directly or indirectly as part of a larger complex of proteins. Although not specifically implicated in morphogenesis in other species, Sgt1 associates with factors important for cell cycle progression [59], [60], which has a fundamental impact on *C. albicans* morphogenesis [32], [61]. In *S. cerevisiae*, Sgt1 is required for both the G1/S and G2/M transitions in the cell cycle, and together with Hsp90, interacts with key cell cycle regulators Skp1 and the Skp-Cullin-F-box (SCF) ubiquitin ligase complex to mediate kinetochore assembly and function [59], [60]. This phenotype is conserved in mammals, where cells lacking Sgt1 experience a delay in mitosis, and Sgt1 and Hsp90 are required for proper kinetochore assembly [62]. In *Drosophila*, Sgt1 stabilizes the mitotic kinase Polo to allow proper centrosome maturation, as well as entry and progression through the cell cycle [63]. Notably, depletion of the Polo-like kinase Cdc5 in *C. albicans* induces Cyr1-dependent filamentous growth [64], suggesting that Sgt1 could similarly function to stabilize Cdc5 in *C. albicans*, as depletion of either protein promotes filamentation. Sgt1 also interacts with Cyr1 in *S. cerevisiae* [45], and Cyr1 is a key morphogenetic regulator in *C. albicans* [9]. Further, in *S. cerevisiae*, a physical interaction between Hsp90 and Sgt1 with Cyr1 plays a key role in regulating gene expression, including genes involved in polarized morphogenesis [65]. We find that Sgt1 interacts with Cyr1 and represses cAMP-dependent PKA activity in *C. albicans* (Figure 3), whereas in *S. cerevisiae* Sgt1 activates, rather than represses, Cyr1-mediated signaling [45], thus reflecting functional divergence in the Sgt1-dependent circuitry between these species. Future studies could test our model that in *C. albicans*, filamentation as well as PKA activation observed upon depletion of Sgt1 is dependent on the adenylyl cyclase Cyr1 by deletion of *CYR1* in the *tetO-SGT1/sgt1*Δ strain.

Sgt1 governs not only *C. albicans* morphogenesis, but also resistance to the azoles and echinocandins (Figures 4 and 5). Though Sgt1 and Hsp90 have a conserved impact on drug resistance phenotypes, the mechanisms involved are distinct. Depletion of Hsp90 leads to destabilization of drug resistance regulator calcineurin [37]; while depletion of Sgt1 does not alter calcineurin stability, it is required for its activation, suggesting a mechanism by which Sgt1 controls antifungal drug resistance (Figure 6). This is reminiscent of drug resistance of *C. albicans* biofilms, where compromising Hsp90 function abrogates resistance of biofilms to azoles, independent of Cna1 stabilization [28]. A similar signature of dependence on Hsp90 for stability and Sgt1 for activation is observed with disease resistance in plants, where depletion of Sgt1 prevents cellular responses associated with Nod1 activation, while having no affect on Nod1 stability, while depletion of Hsp90 results in Nod1 destabilization [66]. While compromising Hsp90 function can block the evolution of drug resistance [35], prior exposure to Hsp90 inhibitors can actually enable the evolution of resistance; pre-treatment with an Hsp90 inhibitor can promote the emergence of azole resistance through the induction of aneuploidies, due to the role of Hsp90 in mediating kinetochore assembly [67]. That Sgt1 is known to act with Hsp90 to mediate kinetochore assembly and function [59], [60], suggests that Sgt1 may also have powerful and opposing effects on the evolution of drug resistance depending on whether the co-chaperone function is compromised prior to or during drug exposure.

Sgt1's capacity to govern morphogenesis, regulate resistance to antifungal drugs, and influence fitness of *C. albicans* in a murine model, suggests that it may provide a promising therapeutic target for fungal infections. Targeting Sgt1 may provide a novel strategy to compromise function of the Hsp90 chaperone machine, as depletion of Sgt1 or Hsp90 has conserved phenotypic effects. Targeting Hsp90 has emerged as powerful strategy for the treatment of fungal infections; compromising Hsp90 function enhances the efficacy of azoles and echinocandins against *C. albicans* in metazoan models of infection [36]. While Hsp90 inhibitors in clinical trials as anti-cancer agents are effective at transforming azoles from ineffective to highly efficacious in a mammalian model of biofilm infection where the drug and infection are localized [28], there is toxicity associated with systemic exposure to Hsp90 inhibitors in a mammalian model of disseminated fungal infection [36]. This toxicity is likely attributed to the deleterious effects of inhibiting a key regulator of signaling and immune responses in the host [68]. A challenge associated with developing fungal selective Hsp90 inhibitors is the high degree of conservation between fungal and human Hsp90 (69% identity and 83% similarity for *S. cerevisiae* [69]). In contrast, fungal Sgt1 bears remarkably less similarity to human Sgt1 (26% identity and 30% similarity for *S. cerevisiae* [44]), potentially facilitating development of fungal-selective inhibitors. The therapeutic potential of targeting Sgt1 is poised to extend to other eukaryotic pathogens for which targeting Hsp90 has therapeutic potential, including the filamentous fungus *Aspergillus fumigatus* [36], the malarial parasite *Plasmodium falciparum* [70], and the African sleeping sickness agent, *Trypanosoma evansi* [71]. Sgt1 provides a powerful strategy for the development of inhibitors targeting the Hsp90 chaperone machine, with broad implications for the treatment of diverse infectious diseases, neurodegenerative disease, and cancer.

## Materials and Methods

### Ethics Statement

All procedures were approved by the Institutional Animal Care and Use Committee (IACUC) at Duke University according to the guidelines of the Animal Welfare Act, The Institute of Laboratory Animal Resources Guide for the Care and Use of Laboratory Animals, and Public Health Service Policy.

### Strains and Culture Conditions

Strains used in this study are listed in Table S1 and their construction is described in the Text S1. *C. albicans* strains were cultured on YPD (2% bacto-peptone, 1% yeast extract, and 2% glucose) or YPM (2% bacto-peptone, 1% yeast extract, and 2% maltose). Overnight cultures were grown in 5 ml of YPD or YPM at 30°C unless otherwise indicated. For doxycycline treatment, cells were grown overnight in 20 µg/mL DOX, subcultured to OD600 of 0.2, and grown in fresh medium with 20 µg/mL DOX and growth until mid-log phase (DOX, BD Biosciences). For glucose-mediated depletion, cells were grown overnight in 50% YPM and 50% YPD, subcultured to OD600 of 0.2, and grown in fresh medium consisting of 50% YPM and 50% YPD and growth until mid-log phase.

### Plasmids

Plasmids used in this study are listed in Table S2 and their construction is described in the Supplemental Methods. Plasmids were sequenced to verify the absence of any nonsynonymous mutations. Primers used in this study are listed in Table S3.

### qRT-PCR

To monitor *SGT1* transcript levels, cells were grown overnight in YPD at 30°C, diluted to OD_600_ of 0.2, and grown overnight again with or without doxycycline, as indicated. Cells were again diluted to OD_600_ of 0.2 in the same conditions and grown to mid-log phase. To monitor *UTR2* transcript levels in response to fluconazole (FL, Sequoia Research Products) treatment, cells were grown overnight in YPD at 30°C, diluted to OD_600_ of 0.2 in YPD with or without 20 µg/ml of doxycycline (DOX) and grown overnight at 30°C. Cells were diluted again to OD_600_ of 0.3 in YPD with or without 20 µg/ml of doxycycline (DOX) and grown for 2 hours at 30°C. After 2 hours, cells were either untreated, treated with 16 µg/ml of fluconazole (FL), or treated with 30 ng/ml micafungin (MF) and grown for an additional 3 hours. Cultures were then pelleted at 1308 rcf for 5 min and frozen overnight at –80°C. RNA was isolated using the QIAGEN RNeasy kit and RNasefree DNase (QIAGEN), and cDNA synthesis was performed using the AffinityScript Multi Temperature cDNA Synthesis Kit (Agilent Technologies). PCR was performed using Fast SYBR® Green Master Mix (Applied Biosystems) and the StepOnePlus Real-Time PCR System (Applied Biosystems) with the following cycling conditions: 95°C for 20 s; then 95°C for 3 min and 60°C for 30 s, for 40 cycles. Reactions were performed in triplicate, with oLC752 and oLC753 (*GPD1*); oLC2030 and oLC2031 (*SGT1*); and oLC1434 and oLC1435 (*UTR2*). Data were analyzed with StepOne™ Software v2.2 (Applied Biosystems).

### Immunoprecipitation

Immunoprecipitations were performed essentially as described [37]. Briefly, yeast cultures were grown overnight in YPD at 30°C. Cells were diluted to OD_600_ of 0.2 in 40 ml and grown to mid-log phase. Cells were washed with sterile H_2_0 and resuspended in 500 µl of lysis buffer containing 20 mM Tris pH 7.5, 100 mM KCl, 5 mM MgCl and 20% glycerol, with one protease inhibitor cocktail (complete, EDTA-free tablet, Roche Diagnostics) per 10 ml, 1 mM PMSF (EMD Chemicals) and 20 mM sodium molybdate (Sigma Aldrich Co.) added fresh before use. Cells were transferred to a 2 ml screw-cap tube and the tube was filled with acid-washed glass beads and additional lysis buffer until the beads were just below the meniscus at the top of the tube to reduce foaming during bead beating. Cells were disrupted by bead beating twice for 4 minutes with a 5-minute break on ice between cycles. Lysates were recovered by piercing a hole in the bottom of each tube, placing each tube in a larger tube, and centrifuging at 1308 rcf for two 5-minute cycles at 4°C, recovering the lysates at each interval. Total collected lysates were cleared by centrifugation at 15,339 rcf for 10 minutes at 4°C and protein concentrations were determined by Bradford analysis.

Anti-IgG immunoprecipitations were done using rabbit IgG agarose (Sigma Aldrich Co.). Protein was diluted to 1 mg/ml in lysis buffer with 0.2% Tween-20, and incubated rotating at 4°C overnight with IgG agarose that had been washed three times with lysis buffer prior to use. Unbound material was removed by washing three times with 1 ml lysis buffer with 0.1% Tween-20, centrifuging at 78 rcf for 5 minutes at 4°C. Protein sample was then incubated with AcTEV Protease (Invitrogen), rotating for 3 hours at 4°C. Protease-treated samples were centrifuged at 78 rcf for 5 minutes at 4°C, and the supernatant was added to Ni-NTA agarose (QIAGEN), that had been washed three times with lysis buffer prior to use. Protein was incubated with Ni-NTA agarose, rotating for 2 hours at 4°C. Samples were then centrifuged at 2817 rcf for 1 minute at 4°C, and protein was eluted by boiling the sample in one volume of 6X sample buffer, containing 0.35 M Tris-HCl, 10% (w/v) SDS, 36% glycerol, 5% β-mercaptoethanol, and 0.012% bromophenol blue.

Anti-HA immunoprecipitations were done using the ProFound^TM^ HA Tag IP/Co-IP Kit (Thermo Scientific). Briefly, protein was diluted to 600 µg in 850 µl of lysis buffer and incubated with 6 µl of anti-HA agarose slurry, rotating overnight at 4°C. Unbound material was removed by washing three times with tris buffered saline containing 0.05% Tween-20 (TBST), pulse centrifuging for 10 seconds. Protein was eluted by boiling in 25–100 µl of 2× non-reducing sample buffer. 2-mercaptoethanol was added after boiling.

Anti-nickel immunoprecipitations (to immunoprecipitate the His tag) were done using Ni-NTA agarose (QIAGEN). Protein was diluted to 1 mg/ml in lysis buffer with 0.2% Tween-20, and incubated rotating at 4°C overnight with Ni-NTA agarose that had been washed three times with lysis buffer prior to use. Unbound material was removed by washing three times with 1 ml lysis buffer with 0.1% Tween-20, centrifuging at 2817 rcf for 1 minute at 4°C. Protein was eluted by incubating with 250 mM imidazole for 5 minutes on ice. Eluted sample was removed from Ni-NTA agarose following centrifugation at 2415 rcf for 30 seconds at room temperature, and 2X sample buffer, containing 2-mercaptoethanol was added and the sample was boiled for 5 minutes.

### Immune Blot Analysis

Immune blot analysis was performed essentially as described [53]. Protein was obtained either from immunoprecipitation protocols above, or alternatively, cells were grown overnight in YPD at 30°C, diluted to OD_600_ of 0.2, and grown overnight again with or without doxycycline, as indicated, then cells were subcultured into fresh medium with or without doxycycline and grown until mid-log phase; at this point, they were harvested by centrifugation and washed with sterile water. Cell pellets were resuspended in lysis buffer containing 50 mM HEPES pH 7.4, 150 mM NaCl, 5 mM EDTA, 1% Triton X-100, 1 mM PMSF, and protease inhibitor cocktail (complete, EDTA-free tablet, Roche Diagnostics). Cells suspended in lysis buffer were mechanically disrupted by adding acid-washed glass beads and bead beating for 3 minutes. Protein concentrations were determined by Bradford analysis. Protein samples were mixed with one-sixth volume of 6X sample buffer for SDS-PAGE. Samples were boiled for 5 minutes and then separated by SDS-PAGE using an 8–10% acrylamide gel. Proteins were electrotransferred to PVDF membranes (Bio-Rad Laboratories, Inc.) and blocked with 5% skimmed milk in phosphate buffered saline (PBS) with 0.1% tween. Blots were hybridized with antibodies against CaHsp90 (1∶10000, generously provided by Brian Larsen [72]), actin (1∶1000 dilution, Santa Cruz Biotechnology, Inc), TAP (1∶5000, Open Biosystems), HA (1∶10, 12CA5, generously provided by Dr. Alan Cochrane), cMyc (1∶50, 9E10, generously provided by Dr. Alan Cochrane).

### Microscopy

Imaging of cells cultured in liquid media was performed using Differential Interference Contrast (DIC) microscopy using a Zeiss Axio Imager. MI and Axiovision software (Carl Zeiss, Inc.). All images were taken with a 100X magnification objective. Imaging of individual colonies on solid media was performed using a Zeiss Stereo Discovery.V8. All images were taken at 8X magnification. Imaging of entire solid media plates was performed using a Canon PowerShot A640 digital camera.

### Invasive Growth

Overnight cultures were grown in 5 ml of YPD with continuous shaking at 30°C. Cells were counted using a hemocytometer, and ∼100 cells were plated onto each YPD agar plate and allowed to grow for 3 days at 37°C. Plates were then washed with water. Plates for photographed before and after washing using a Canon PowerShot A640 camera.

### Protein Kinase A Activity Assay

Cells were grown overnight in YPD at 30°C, diluted to OD_600_ of 0.2, and grown overnight again with or without doxycycline, as indicated. Cells were again diluted to OD_600_ of 0.2 in the same conditions, in 10 ml and grown to mid-log phase. Cells were pelleted and washed with 5 ml of 1X phosphate-buffered saline (PBS). Cells were resuspended in 200 µl of PKA extraction buffer (25 mM Tris-HCl pH 7.4, 0.5 mM EDTA, 0.5 mM EGTA, 10 mM 2-mercaptoethanol, 1 µg/ml leupeptin and 1 µg/ml aprotinin) and mechanically disrupted by adding acid-washed glass beads and bead beating for 3 minutes. Protein concentrations were determined by Bradford analysis. PKA activity was established essentially as described [50]. Briefly, 10 µg of total protein was used, and PKA activity was measured following the guidelines of the PepTag® Assay for Non-Radioactive Detection of cAMP-Dependent Protein Kinase (Promega) in a total volume of 25 µl, 20 mM Tris-HCl pH 7.4, 10 mM MgCl_2_, 1 mM ATP, 2 µg of PepTag® A1 Peptide and 10 µM cAMP. Reactions were incubated at 30°C for 30 minutes, inactivated at 95°C for 10 minutes and separated on a 0.8% agarose gel made with 50 mM Tris-HCl pH 8.0, run at 100 V for 15 minutes. Quantification of the phosphorylated peptide fractions excised from the gels was performed by spectrophotometry in a 96-well plate at 570 nm.

### Minimum Inhibitory Concentration Assays

Minimum inhibitory concentration (MIC) assays were performed in flat-bottom, 96-well microtiter plates (Sarstedt) using a modified broth microdilution protocol, essentially as described [35]. MIC tests were set up in a total volume of 0.2 ml/well with two-fold dilutions of fluconazole in YPD (FL, Sequoia Research Products) or micafungin (MF, generously provided by Julia R. Köhler). Gradients of fluconazole were from 256 µg/ml down to 0 with the following concentration steps in µg/ml: 256, 128, 64, 32, 16, 8, 4, 2, 1, 0.5, 0.25, 0, and gradients of micafungin were from 2 or 8 µg/ml down to 0 with the following concentration steps in µg/ml: 8, 4, 2, 1, 0.5, 0.25, 0.125, 0.0625, 0.03125, 0.015625, 0.0078125, 0 or 2, 1, 0.5, 0.25, 0.125, 0.0625, 0.03125, 0.015625, 0.0078125, 0.00390625, 0.001953125, 0. Doxycycline was included in plates at a constant concentration of 20 µg/ml, as indicated. Strains were grown overnight in YPD at 30°C. Cell densities of overnight cultures were determined and dilutions were prepared such that ∼10^3^ cells were inoculated into each well. Plates were incubated in the dark at 30°C for 48 hours or 72 hours, as indicated, at which point plates were sealed and re-suspended by agitation. Absorbance was determined at 600 nm using a spectrophotometer (Molecular Devices) and was corrected for background from the corresponding medium. Each strain was tested in duplicate on at least two occasions. MIC data were quantitatively displayed with colour using the program Java TreeView 1.1.3 (http://jtreeview.sourceforge.net).

### Cidality Assays

Cidality was established essentially as previously described [38]. Briefly, MIC assays were performed as described above, and incubated at 30°C for 48 hours or 72 hours, as indicated. Cells from the MIC assay were then spotted onto solid YPD medium using a spotter, incubated at 30°C for 48 hours, and photographed using a Canon PowerShot A640 digital camera.

### Murine Model of *C. albicans* Infection

To prepare inoculum, cultures of wild type or *tetO-SGT1/sgt1*Δ were started from frozen stocks onto Sabouraud dextrose agar plates and incubated at 35°C for 48 hours. Colonies were suspended in sterile pH 7.4 phosphate-buffered saline (PBS), centrifuged at 324 rcf for 5 minutes, washed with sterile PBS one time and diluted to the desired concentration (2.1×10^6^ cells/ml) as verified by counting on a Neubauer hematocytometer. Concentration was verified by serial dilution and culture on Saboraud dextrose plates. For murine exposure, male CD1 mice (Charles River Laboratories, Wilmington, MA) age 8 weeks (weight 28–34 g) were infected via the tail vein with 100 µl of a 2.1×10^6^ CFU/ml of yeast suspension. For the wild-type strain SN95 (CaLC239) the sample size was *n* = 11 for the untreated and *n* = 12 for the tetracycline treated, for the *tetO-SGT1/sgt1*Δ strain (CaLC1966) the sample size was *n* = 11 in each of the treated and untreated groups. Mice receiving tetracycline were given sterile water supplemented with 2 mg/ml of tetracycline hydrochloride (Calbiochem) beginning 7 days prior to infection and continued until sacrifice. Water was changed once daily. Daily weights during the 7-day lead in period were taken to ensure that mice remained hydrated. Mice were observed three times daily for signs of illness and weighed daily. At day 3 after injection, mice were sacrificed by CO_2_ asphyxiation and the left kidney was removed aseptically, collected in 1 ml of PBS and homogenized for 50 seconds in a mini Bead beater (BioSpec) using 0.5 zirconia and silica beads. 10-fold serial dilutions were plated for determination of kidney fungal burden. CFU values in kidneys were expressed as CFU/g of tissue, log-transformed and compared using a Kruskal-Wallis test with Dunn's Multiple Comparison post-test (GraphPad Prism 5.0). All murine work was performed under a protocol approved by the Institutional Animal Use and Care Committee at Duke University Medical Center.

## Supporting Information

Figure S1 **Genetic depletion of Cdc37 does not induce yeast to filament morphogenesis, while genetic depletion of Sgt1 induces morphogenesis.** (A) Genetic depletion of Cdc37, upon growth of the *MAL2p-CDC37/cdc37*Δ strain in the presence of glucose, does not alter yeast morphology. Cdc37 levels were reduced by growth in rich medium containing 1% glucose and 1% maltose in the *MAL2p-CDC37/cdc37*Δ strain at 30°C for 24 hours, as indicated. (B) Genetic depletion of Sgt1 induces filamentation. Sgt1 levels were reduced by growth overnight in 20 µg/ml doxycycline, followed by subculture in fresh medium with 20 µg/ml doxycycline and growth until mid-log phase.
(TIF)

Figure S2 **Characterization of SGT1 depletion.** (A) Growth of triplicate samples was monitored at 30°C by spectrophotometer every 15 minutes over 48 hours with continuous agitation, in sealed 96-well plates. *SGT1/SGT1, SGT1/sgt1Δ,* and *tetO-SGT1/sgt1Δ* strains were grown with 2.5 μg/ml –20 μg/ml doxycycline (DOX), as indicated. (B) Despite the reduced growth rates in (A), which reflects an environment with reduced oxygen availability due to the seal, transcriptional repression of *SGT1* causes no reduction in stationary phase density reached in cultures grown in well-aerated tubes with continuous agitation, and only a minor reduction in growth when Sgt1 levels were reduced by growth overnight in 20 µg/mL DOX, followed by subculture in fresh medium with 20 µg/mL DOX and growth until mid-log phase, the conditions used for most assays. Optical density (OD600) was measured following growth overnight with or without DOX, and after growth until mid-log, with or without DOX, as indicated. (C) Cells remain viable after transcriptional repression of *SGT1* in cultures grown in well-aerated tubes with continuous agitation when Sgt1 levels were reduced by growth overnight in 20 µg/mL DOX, followed by subculture in fresh medium with 20 µg/mL DOX and growth until mid-log phase, the conditions used for most assays. Cells were plated on to YPD following growth overnight with or without DOX, and after growth until mid-log, with or without DOX, as indicated, and colony forming units (CFUs) were counted.
(TIF)

Figure S3 **Hsp90 client protein Cna1 retains stability upon depletion of Sgt1.** (A) Cna1 is destabilized upon depletion of Hsp90. Hsp90 levels were reduced by growth overnight in 50% YPM and 50% YPD, followed by subculture in fresh medium consisting of 50% YPM and 50% YPD and growth until mid-log phase. First panel, immune blot analysis of Hsp90 levels (5 µg protein loaded per well); and second panel, immune blot analysis of Cna1-TAP levels (50 µg protein loaded per well). Actin was used as a loading control. (B) Cna1 retains stability upon depletion of Sgt1. Sgt1 levels were reduced by growth overnight in 50% YPM and 50% YPD, followed by subculture in fresh medium consisting of 50% YPM and 50% YPD and growth until mid-log phase. First panel, immune blot analysis of Sgt1 levels (50 µg protein loaded per well); and second panel, immune blot analysis of Cna1-TAP levels (50 µg protein loaded per well). Actin was used as a loading control.
(TIF)

Table S1 **Strains used in this study.**
(DOC)

Table S2 **Plasmids used in this study.**
(DOC)

Table S3 **Oligonucleotides used in this study.**
(DOC)

Text S1 **Supporting Materials and Methods.**
(DOC)
