# Supplementary figures and images for: The Hsp90 Co-Chaperone Sgt1 Governs Candida albicans Morphogenesis and Drug Resistance
Source: PLoS One. 2012 Sep 6;7(9):e44734. doi: 10.1371/journal.pone.0044734 (PMC3435277; doi:10.1371/journal.pone.0044734)

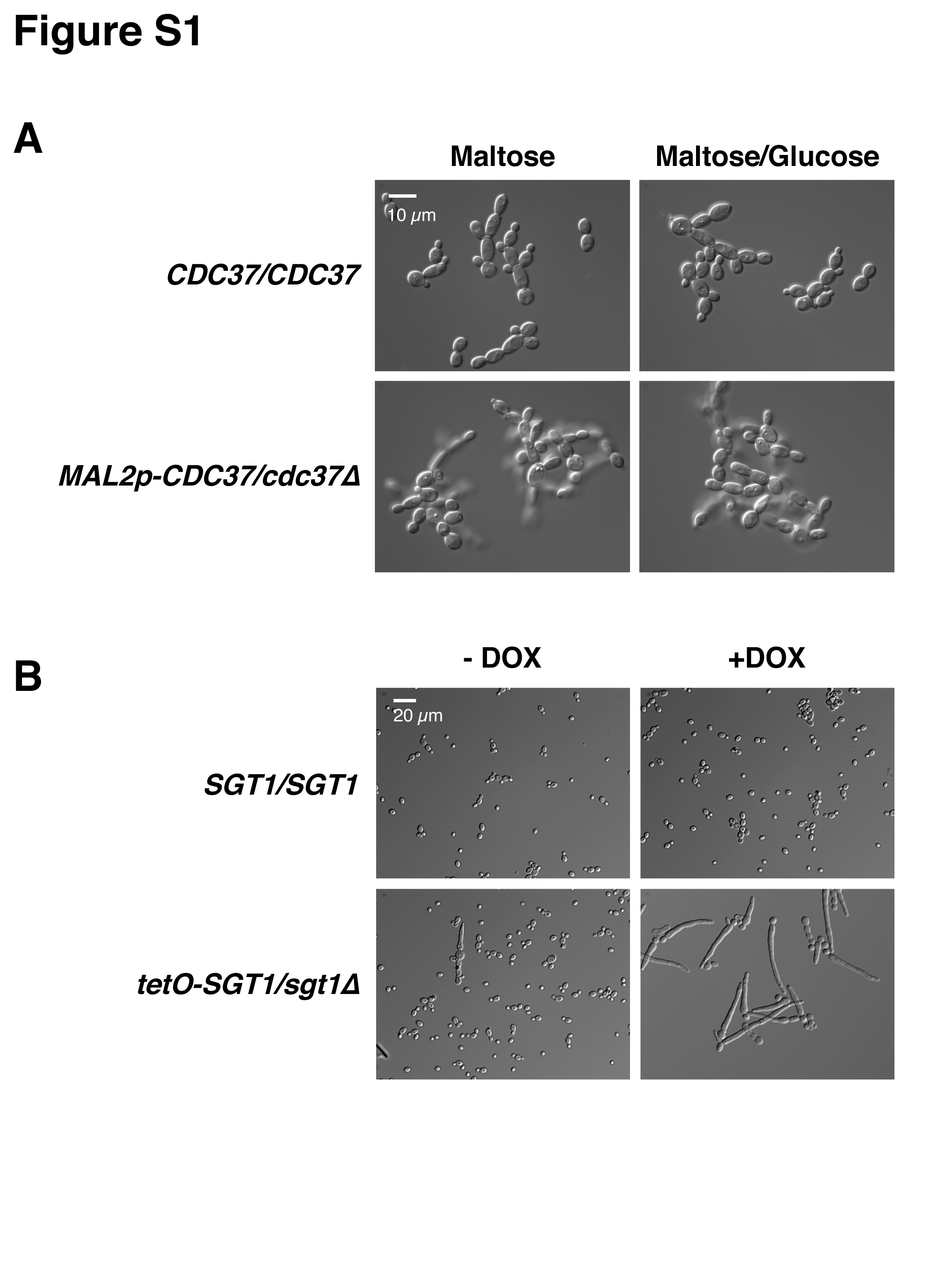

Supplement: Figure S1 — Genetic depletion of Cdc37 does not induce yeast to filament morphogenesis, while genetic depletion of Sgt1 induces morphogenesis. (A) Genetic depletion of Cdc37, upon growth of the MAL2p-CDC37/cdc37Δ strain in the presence of glucose, does not alter yeast morphology. Cdc37 levels were reduced by growth in rich medium containing 1% glucose and 1% maltose in the MAL2p-CDC37/cdc37Δ strain at 30°C for 24 hours, as indicated. (B) Genetic depletion of Sgt1 induces filamentation. Sgt1 levels were reduced by growth overnight in 20 µg/ml doxycycline, followed by subculture in fresh medium with 20 µg/ml doxycycline and growth until mid-log phase. (TIF) [file pone.0044734.s001.tif]

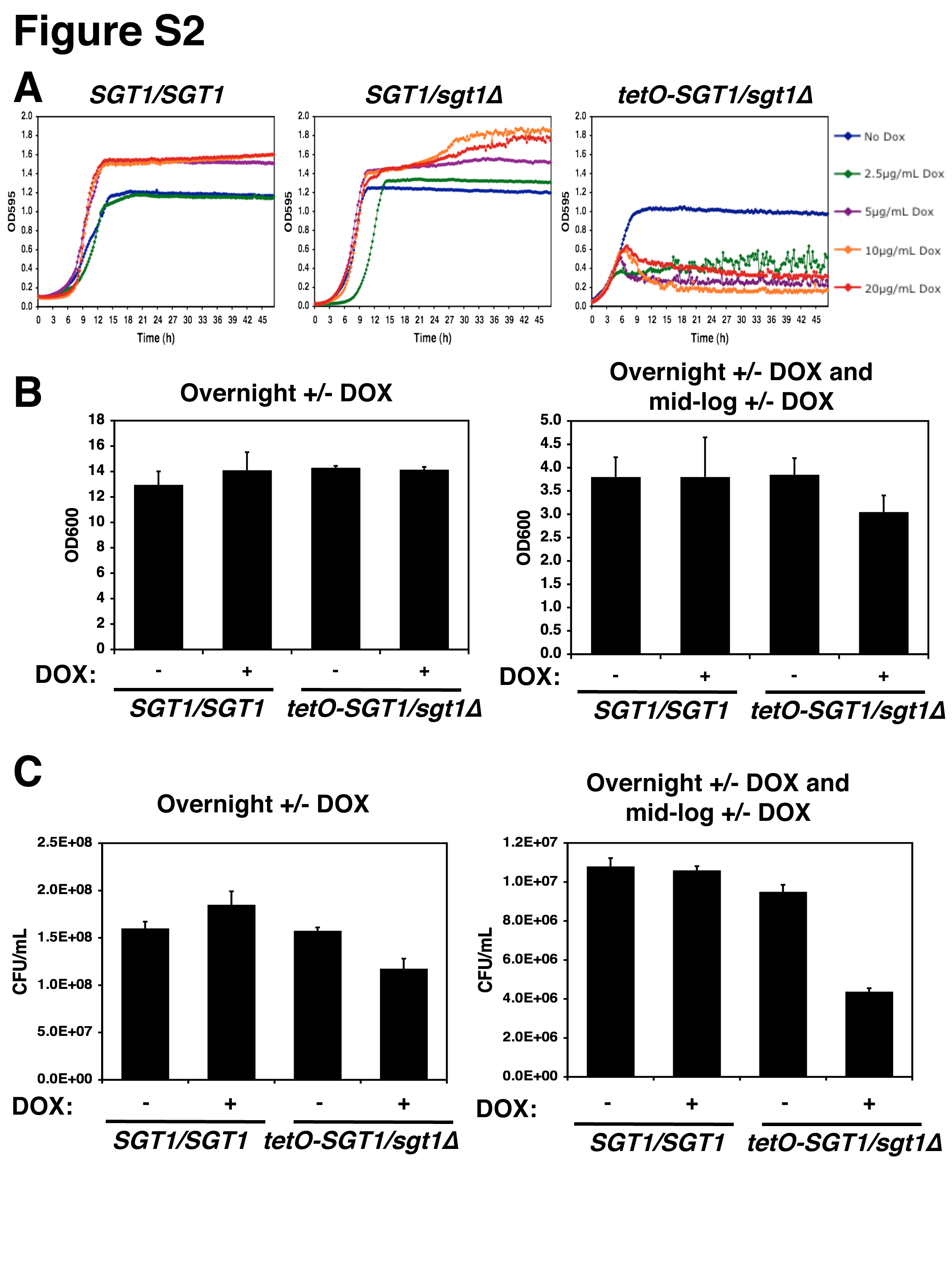

Supplement: Figure S2 — Characterization of SGT1 depletion. (A) Growth of triplicate samples was monitored at 30°C by spectrophotometer every 15 minutes over 48 hours with continuous agitation, in sealed 96-well plates. SGT1/SGT1, SGT1/sgt1Δ, and tetO-SGT1/sgt1Δ strains were grown with 2.5 μg/ml –20 μg/ml doxycycline (DOX), as indicated. (B) Despite the reduced growth rates in (A), which reflects an environment with reduced oxygen availability due to the seal, transcriptional repression of SGT1 causes no reduction in stationary phase density reached in cultures grown in well-aerated tubes with continuous agitation, and only a minor reduction in growth when Sgt1 levels were reduced by growth overnight in 20 µg/mL DOX, followed by subculture in fresh medium with 20 µg/mL DOX and growth until mid-log phase, the conditions used for most assays. Optical density (OD600) was measured following growth overnight with or without DOX, and after growth until mid-log, with or without DOX, as indicated. (C) Cells remain viable after transcriptional repression of SGT1 in cultures grown in well-aerated tubes with continuous agitation when Sgt1 levels were reduced by growth overnight in 20 µg/mL DOX, followed by subculture in fresh medium with 20 µg/mL DOX and growth until mid-log phase, the conditions used for most assays. Cells were plated on to YPD following growth overnight with or without DOX, and after growth until mid-log, with or without DOX, as indicated, and colony forming units (CFUs) were counted. (TIF) [file pone.0044734.s002.tif]

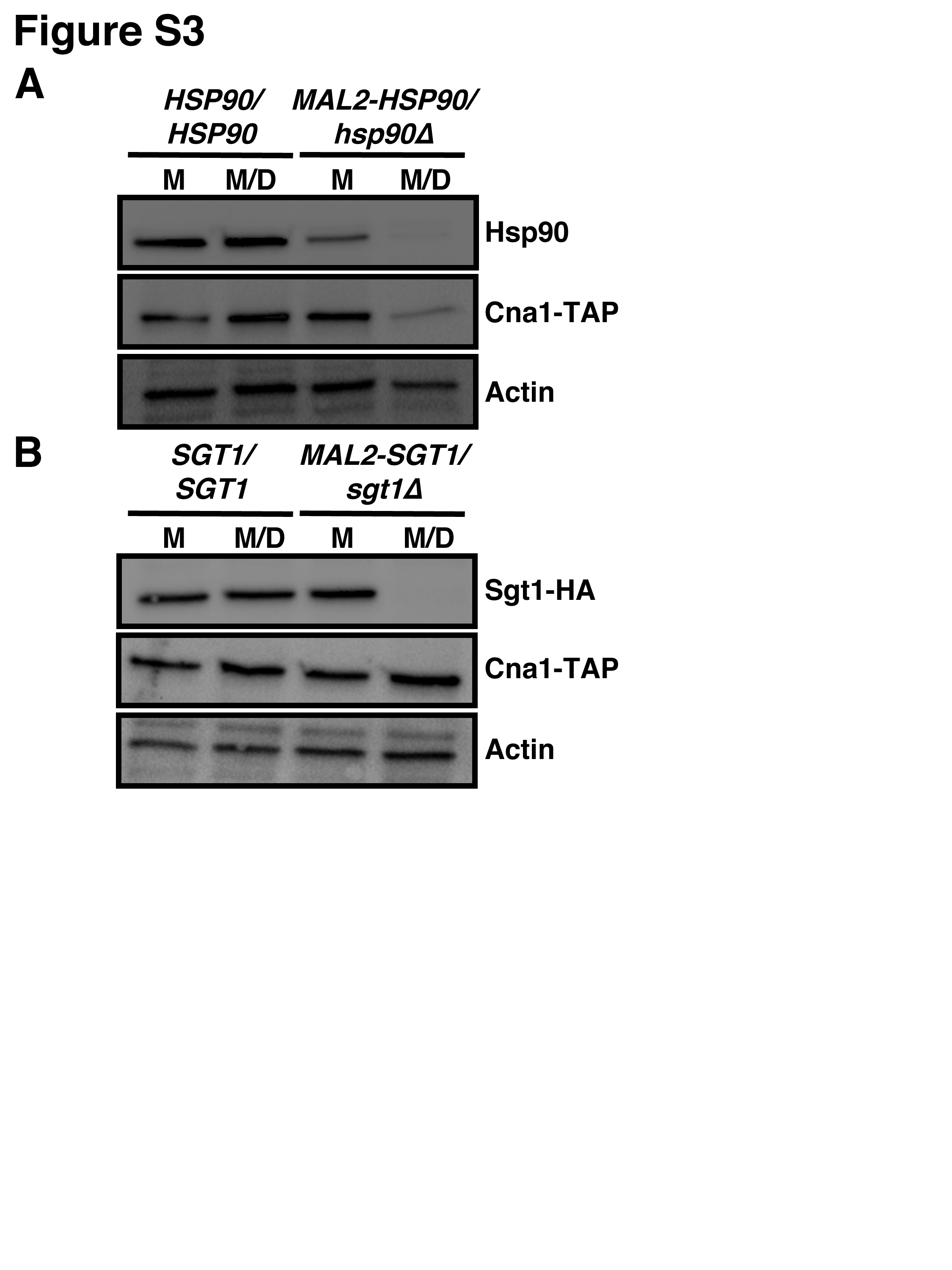

Supplement: Figure S3 — Hsp90 client protein Cna1 retains stability upon depletion of Sgt1. (A) Cna1 is destabilized upon depletion of Hsp90. Hsp90 levels were reduced by growth overnight in 50% YPM and 50% YPD, followed by subculture in fresh medium consisting of 50% YPM and 50% YPD and growth until mid-log phase. First panel, immune blot analysis of Hsp90 levels (5 µg protein loaded per well); and second panel, immune blot analysis of Cna1-TAP levels (50 µg protein loaded per well). Actin was used as a loading control. (B) Cna1 retains stability upon depletion of Sgt1. Sgt1 levels were reduced by growth overnight in 50% YPM and 50% YPD, followed by subculture in fresh medium consisting of 50% YPM and 50% YPD and growth until mid-log phase. First panel, immune blot analysis of Sgt1 levels (50 µg protein loaded per well); and second panel, immune blot analysis of Cna1-TAP levels (50 µg protein loaded per well). Actin was used as a loading control. (TIF) [file pone.0044734.s003.tif]
