# Supplementary material for: The Hsp90 Co-Chaperone Sgt1 Governs Candida albicans Morphogenesis and Drug Resistance
Source: PLoS One. 2012 Sep 6;7(9):e44734. doi: 10.1371/journal.pone.0044734 (PMC3435277; doi:10.1371/journal.pone.0044734)
Supplement: Table S1 — Strains used in this study. (DOC) [file pone.0044734.s004.doc]

**Table S1: Strains used in this study.**

| **Strain Name** | **Alias** | **Genotype** | **Source** |
| --- | --- | --- | --- |
| CaLC239 | SN95 | *arg4∆/arg4∆ his1∆/his1∆ URA3/ura3::imm434 IRO1/iro1::imm434* | [4] |
| CaLC206 |  | As SN95, *his1∆/his1::tetR-FRT* | [11] |
| CaLC1970 |  | As SN95, *his1∆/his1:: tetR-FRT SGT1/sgt1::FRT* | This study |
| CaLC1966 |  | As SN95, *his1∆/his1:: tetR-FRT FRT-tetO-SGT1/sgt1::FRT* | This study |
| CaLC1779 |  | As SN95, *SGT1/SGT-HA::FRT* | This study |
| CaLC501 |  | As SN95, *HSP90/HSP90-TAP::FRT* | [5] |
| CaLC1759 |  | As SN95, *HSP90/HSP90-TAP::FRT SGT1/SGT-HA::FRT* | This study |
| CaLC718 | 1003 | *MET3p-HFM-CYR1-URA3/cyr1 ::ARG4 RAS1/GFP-RAS1-HIS1* | [6] |
| CaLC1793 |  | As CaLC1793, *SGT1/SGT-HA::FRT* | This study |
| CaLC1411 | CaLC436 | As SN95, *his1∆/his1:: tetR-FRT FRT-tetO-HSP90/hsp90::CdHIS1* | [5] |
| CaLC660 |  | As SN95, *his1∆/his1:: tetR-FRT erg3::FRT/erg3::FRT* | [7] |
| CaLC2110 |  | As SN95, *his1∆/his1:: tetR-FRT FRT-tetO-SGT1/sgt1::FRT erg3::FRT/erg3::FRT* | This study |
| CaLC2087 |  | As SN95, *FKS1/FKS1 T1917C;T1922C* | This study |
| CaLC2112 |  | As SN95, *his1∆/his1:: tetR-FRT FRT-tetO-SGT1/sgt1::FRT FKS1/FKS1 1T1917C;T1922C* | This study |
| CaLC2286 |  | As SN95, *SGT1-HA::FRT/SGT1 CNA1/CNA1-TAP::HIS1* | This study |
| CaLC2309 |  | As SN95, *his1∆/his1:: tetR-FRT FRT-tetO-SGT1-HA::FRT/sgt1::FRT CNA1/CNA1-TAP::HIS1* | This study |
| CaLC2276 |  | As SN95, *CNA1/CNA1-TAP::ARG4* | This study |
| CaLC2278 |  | As SN95, *his1∆/his1:: tetR-FRT FRT-MAL2p-HSP90/hsp90::CdHIS1 CNA1/CNA1-TAP::ARG4* | This study |
| CaLC2310 |  | As SN95, *his1∆/his1:: tetR-FRT FRT-MAL2p-SGT1-HA::FRT/sgt1::FRT CNA1/CNA1-TAP::HIS1* | This study |
| CaLC432 |  | As SN95, *his1∆/his1:: tetR-FRT FRT-MAL2p-HSP90::FRT/hsp90:: CdHIS1* | [5] |
