# Supplementary material for: The Hsp90 Co-Chaperone Sgt1 Governs Candida albicans Morphogenesis and Drug Resistance
Source: PLoS One. 2012 Sep 6;7(9):e44734. doi: 10.1371/journal.pone.0044734 (PMC3435277; doi:10.1371/journal.pone.0044734)
Supplement: Table S2 — Plasmids used in this study. (DOC) [file pone.0044734.s005.doc]

**Table S2: Plasmids used in this study.**

| **Plasmid Name** | **Alias** | **Description (Backbone)** | **Source** |
| --- | --- | --- | --- |
| pLC49 | pJK863 | *FLP-NAT*, ampR | [3] |
| pLC330 |  | *tetO-CaHSP90*, *NAT*, ampR (pLC49) | [5] |
| pLC624 |  | *FLP-NAT-tetO-SGT1*, ampR | This study |
| pLC625 |  | *FLP-NAT-MAL2-SGT1*, ampR | This study |
| pLC583 |  | *SGT1-HA-FLP-NAT*, ampR | This study |
| pLC361 |  | *FLP-NAT-ERG3*-KO, ampR | [7] |
| pLC572 | FJ160457 | pFA-*TAP-HIS1*, ampR | [9] |
| pLC573 | FJ160458 | pFA-*TAP-ARG4*, ampR | [9] |
| pLC90 | pAU22 | *MAL2p-lacZ*, *URA3*, ampR | [10] |
| pLC332 |  | *FLP-NAT-MAL2p*, ampR | This study |
| pLC506 |  | *FLP-NAT-MAL2p-CDC37*, ampR | This study |
| pLC505 |  | *FLP-NAT-CDC37*-KO, ampR | This study |
