# Supplementary material for: The Hsp90 Co-Chaperone Sgt1 Governs Candida albicans Morphogenesis and Drug Resistance
Source: PLoS One. 2012 Sep 6;7(9):e44734. doi: 10.1371/journal.pone.0044734 (PMC3435277; doi:10.1371/journal.pone.0044734)
Supplement: Table S3 — Oligonucleotides used in this study. (DOC) [file pone.0044734.s006.doc]

**Table S3: Oligonucleotides used in this study.**

| **Name** | **Description** | **Sequence** |
| --- | --- | --- |
| oLC1831 | CaSgt1-NATflipper-F | GAAAAATCAGCGTATTAATCACCTCTAACGAACAAGAGTTCACCGTCAAAAAAGAATAAAAAACAAATTAGGAAACAGCTATGACCATG |
| oLC1832 | CaSgt1-NATflipper-R | CATTACCATTCATAAAAAACAAACTGATATTAAACAGCTATACTGCTACTCAACTGAGCTACACACCGTTGTAAAACGACGGCCAG |
| oLC275 | pJK863up-R | AAAGTCAAAGTTCCAAGGGG |
| oLC1839 | CaSgt1-389-F | GGCACATTTGTTGTGTTACC |
| oLC274 | pJK863down-F | CTGTCAAGGAGGGTATTCTGG |
| oLC1840 | CaSgt1+1582-R | CCATCTCATAGGAATTGATTC |
| oLC1885 | TetO-CaSgt1-pLC330-F | GAAAAATCAGCGTATTAATCACCTCTAACGAACAAGAGTTCACCGTCAAAAAAGAATAAAAAACAAATTACGAGGAAGTTCCTATACTTT |
| oLC1925 | CaSgt1+500-SacI-R | CCCGAGCTCGGTTCATGAGATTTGTCCTTC |
| oLC1741 | Sgt1-614-F | GGTATTATTAACGTGGGTGG |
| oLC300 | Tetp-F-NotI | ATAAGAATGCGGCCGCGTTTGGTTCAGCACCTTGTCG |
| oLC1742 | Sgt1+575-R | GGAGCAGATGATGTAGACTC |
| oLC1839 | CaSgt1-389-F | GGCACATTTGTTGTGTTACC |
| oLC1840 | CaSgt1+1582-R | CCATCTCATAGGAATTGATTC |
| oLC1477 | CDC28+776-R | GGAACTGCTTCACTCAAAGG |
| oLC1631 | Sgt1+677-F | CCAATGAGGAAGTTATTATC |
| oLC1632 | Sgt1+1829-R | GGTGTATATGTAACTAATGG |
| oLC499 | CaERG3-513-F | CAAAACTACTTGTTAAGC |
| oLC500 | CaERG3-1683-R | GTTGATGTGATGTAAGTTAG |
| oLC166 | CaERG3-R4 | GCTGGGAAAAATTTAGGAGC |
| oLC2049 | CaFKS1+1877F T1917C T1922C | TATGGTTTTTGGTTTTCCTTGCCAAATTGGTTGAATCTTAcTTCTcCTTGACATTGTCTTTAAGAGATCCTATTAGAAACTTGTCGACCA |
| oLC1605 | CaFKS1+1566-F | AGAATGGGCCGGTGCTCAACA |
| oLC2053 | CaFKS1+2220-R | CGTGGTAGCTAAAATCTTGG |
| oLC1634 | HIS1+206-R | CCTTCTCCAACGAAAACTGG |
| oLC1645 | HIS-F | ACAAACCTACTAATATCAGAT |
| oLC1708 | Ca-CNA1-TAP-R | CTTAACTATATATAAATATATATATATATAGATTATTATTCTTATATAAAAACAGGGGCAAAAGAAAGTTCGATGAATTCGAGCTCGTT |
| oLC2095 | CaCna1+1678-F | GCTGATAGAGTTAATGAAGC |
| oLC2096 | CaCna1+1944-R | GATGATTCAACTCTGACAGG |
| oLC1594 | ARG4-F | ATGTTGGCTACTGATTTAGCTG |
| oLC1593 | TAP-R | TAAACTTTGGATGAAGGCG |
| oLC1594 | ARG4-F | ATGTTGGCTACTGATTTAGCTG |
| oLC1924 | CaSgt1+1-SacII-F | TCCCCGCGGATGGCAATAGAACAATTTATCAC |
| oLC1925 | CaSgt1+500-SacI-R | CCCGAGCTCGGTTCATGAGATTTGTCCTTC |
| oLC301 | Tetp-R-SacII | GGCACCGCGGCGACTATTTATATTTGTATGTGTGTAGG |
| oLC1622 | Sgt1+735-F-KpnI | GGGGTACCGGTCGAATTTGATACAAACTC |
| oLC1623 | Sgt1-HA+1239-R-ApaI | TTGCGGGCCCTCAGGCGGCCGGAGCGTAATCTGGAACGTCATATGGATACGTATCCCATTTCTTGACTTCC |
| oLC1624 | Sgt1+1243-F-SacII | TCCCCGCGGAACGGTGTGTAGCTCAGTTG |
| oLC1625 | Sgt1+1757-R-SacI | CCCGAGCTCGGATTCCCATCCGAATGATC |
| oLC752 | GPD1+570-F | AGTATGTGGAGCTTTACTGGGA |
| oLC753 | GPD1+766-R | CAGAAACACCAGCAACATCTTC |
| oLC2030 | CaSGT1+548-F | CCAACCAAGAGTCTACATCA |
| oLC2031 | CaSGT1+768-R | GGAAATAGAAACCGAGTTTG |
| oLC1089 | CaCDC37-490+Apa1-F | TTGCGGGCCCGGTGAATTCACCCTTGCTGATG |
| oLC1090 | CaCDC37+1+Apa1-R | TTGCGGGCCCGATTGATCTTAATTACAATTTAGG |
| oLC1091 | CaCDC37+1527+SacII-F | TCCCCGCGGATAGCGTAATAAAAATTGCAC |
| oLC1092 | CaCDC37+2008+SacI-R | CCCGAGCTCCAGCACAAATTCAAAGCATG |
| oLC1095 | CaCDC37+1+SacII-F | TCCCCGCGGATGCCAATAGATTACTCCAAG |
| oLC1096 | CaCDC37+505+SacI-R | CCCGAGCTCCGTCGATCCTGTTTCTATGT |
| oLC1093 | CaCDC37-645-F | CCATCTCCAGGTAACAACTC |
| oLC1097 | CaCDC37+662-R | GGAGCTTTTGGTTTATCTTG |
| oLC1094 | CaCDC37+2150-R | CCACAATCTTATGGTCAACC |
| oLC441 | CaMAL2-528-F-NotI | ATAAGAATGCGGCCGCgtctagtaccatctgtacc |
| oLC442 | CaMAL2-483-F-NotI | ATAAGAATGCGGCCGCGTCTAACTCGACTGTCTGG |
| oLC1434 | CaUTR2+136-F | GGTATCTGTGGTACTGGGGC |
| oLC1435 | CaUTR2+394-R | CAACGGTACCAGTGGTATG |
| oLC2308 | CaCNA1-TAP-F | ACAACAAATTGAAAATCAAGAAATGAGTGGACCAGTTTTCCAAAGATTAATAAGAAGATTATCTCAAAGTggtcgacggatccccgggtt |
| oLC2309 | CaCNA1-TAP-R | GCTTAACTATATATAAATATATATATATATAGATTATTATTCTTATATAAAAACAGGGGCAAAAGAAAGTtcgatgaattcgagctcgtt |
