# Supplementary material for: The Hsp90 Co-Chaperone Sgt1 Governs Candida albicans Morphogenesis and Drug Resistance
Source: PLoS One. 2012 Sep 6;7(9):e44734. doi: 10.1371/journal.pone.0044734 (PMC3435277; doi:10.1371/journal.pone.0044734)
Supplement: Text S1 — Supporting Materials and Methods. (DOC) [file pone.0044734.s007.doc]

**Text S1**

**Supporting Materials and Methods**

**Strain Construction**

**CaLC1970:** The *SGT1* knockout construct was PCR amplified from pLC49 using primer pair oLC1831/oLC1832, containing sequence homologous to upstream and downstream regions of *SGT1*, and transformed into CaLC206, containing the tetracycline-repressible transactivator *tetR* at the HIS1 [1]. Nourseothricin (NAT)-resistant transformants were PCR tested for proper integration of the construct using primer pairs oLC275/oLC1839 and oLC274/oLC1840. The *SAP2* promoter was induced to drive expression of FLP recombinase [2,3] to excise the NAT marker cassette.

**CaLC1966:** The *tetO-SGT1* construct was PCR amplified from pLC624, containing NAT, *tetO* and sequence homologous to downstream of *SGT1,* using primer pair oLC1885/oLC1925, containing sequence homologous to upstream of *SGT1.* NAT-resistant transformants were PCR tested for proper integration of the construct using primer pairs oLC1741/oLC275 and oLC300/oLC1742; and were PCR tested using primer pair oLC1741/oLC1742 to verify absence of additional wild-type alleles of *SGT1*, primer pair oLC1839/oLC1840 to verify the presence of the deleted allele of *SGT1*, and primer pair oLC300/oLC1477 to verify the presence of the *tetO-SGT1* allele. The *SAP2* promoter was induced to drive expression of FLP recombinase to excise the NAT marker cassette.

**CaLC1779:** The plasmid pLC583, containing the *SGT1-HA* tagging construct, was digested with BssHII and transformed into CaLC239 (SN95, [4]). NAT-resistant transformants were PCR tested for proper integration of the construct using primer pairs oLC1631/oLC275 and oLC274/oLC1632. The *SAP2* promoter was induced to drive expression of FLP recombinase to excise the NAT marker cassette.

**CaLC1759:** The plasmid pLC583, containing the *SGT1-HA* tagging construct, was digested with BssHII and transformed into CaLC501 [5]. NAT-resistant transformants were PCR tested for proper integration of the construct using primer pairs oLC1631/oLC275 and oLC274/oLC1632. The *SAP2* promoter was induced to drive expression of FLP recombinase to excise the NAT marker cassette.

**CaLC1793:** The plasmid pLC583, containing the *SGT1-HA* tagging construct, was digested with BssHII and transformed into CaLC718 [6]. NAT-resistant transformants were PCR tested for proper integration of the construct using primer pairs oLC1631/oLC275 and oLC274/oLC1632. The *SAP2* promoter was induced to drive expression of FLP recombinase to excise the NAT marker cassette.

**CaLC2110:** The plasmid pLC361 [7], containing the *ERG3* knockout construct, was digested with KpnI and SacI and transformed into CaLC1966. NAT-resistant transformants were PCR tested for proper integration of the construct using primer pairs oLC275/oLC499 and olC274/oLC500. The *SAP2* promoter was induced to drive expression of FLP recombinase to excise the NAT marker cassette. Again, the plasmid pLC361, containing the *ERG3* knockout construct, was digested with KpnI and SacI and transformed into CaLC1966 containing one allele of *ERG3* already deleted. NAT-resistant transformants were PCR tested for proper integration of the construct using primer pairs oLC275/oLC499 and olC274/oLC500. The strain was additionally PCR tested with primer pair oLC499/oLC500 to verify presence of the deleted allele of *ERG3*, and with primer pair oLC499/oLC166 to verify the absence of any wild-type alleles of *ERG3*. The *SAP2* promoter was induced to drive expression of FLP recombinase to excise the NAT marker cassette.

**CaLC2087:** The strain CaLC239 was transformed with 50 µg of the oLC2049 oligo, containing *FKS1* sequence, a mutation that imparts echinocandin resistance (T1922C) [8], as well as a silent mutation (T1917C). Transformants resistant to the echinocandins were selected on plates containing 2 µg/ml micafungin. Resistant transformants were sequenced to verify presence of both mutations. For sequencing, a portion of *FKS1* was amplified from the resistant colonies by PCR using primer pair oLC1605/oLC2053. The PCR product was purified and sequenced with oLC1605.

**CaLC2112:** The strain CaLC1966 was transformed with 50 µg of the oLC2049 oligo, containing *FKS1* sequence, a mutation that imparts echinocandin resistance (T1922C) [8], as well as a silent mutation (T1917C). Transformants resistant to the echinocandins were selected on plates containing 2 µg/ml micafungin. Resistant transformants were sequenced to verify presence of both mutations. For sequencing, a portion of *FKS1* was amplified from the resistant colonies by PCR using primer pair oLC1605/oLC2053. The PCR product was purified and sequenced with oLC1605.

**CaLC2132:** The plasmid pLC583, containing the *SGT1-HA* tagging construct, was digested with BssHII and transformed into CaLC2108. NAT-resistant transformants were PCR tested for proper integration of the construct using primer pairs oLC300/oLC275 to verify the tagging construct integrated at the *tetO* locus, with primer pair and oLC274/oLC1632. The strain was additionally tested with primer pair oLC300/oLC1632 to verify the absence of additional wild-type alleles of *SGT1*, and with oLC1741/oLC1742 to confirm presence of the deleted allele of *SGT1*.

**CaLC2133:** The plasmid pLC583, containing the *SGT1-HA* tagging construct, was digested with BssHII and transformed into CaLC2109. NAT-resistant transformants were PCR tested for proper integration of the construct using primer pairs oLC300/oLC275 to verify the tagging construct integrated at the *tetO* locus, with primer pair and oLC274/oLC1632. The strain was additionally tested with primer pair oLC300/oLC1632 to verify the absence of additional wild-type alleles of *SGT1*, and with oLC1741/oLC1742 to confirm presence of the deleted allele of *SGT1*.

**CaLC1376:** The plasmid pLC506 was digested with BssHII liberate the *MAL2p-CDC37* cassette, and was transformed into CaLC239. NAT-resistant transformants were PCR tested for proper integration of the construct using primer pairs oLC1093/oLC275 and oLC274/oLC1097. The *SAP2* promoter was induced to drive expression of FLP recombinase to excise the NAT marker cassette. The plasmid pLC505 was then digested with BssHII to liberate the *CDC37* knockout cassette, and transformed into the strain containing *MAL2p-CDC37*. NAT-resistant transformants were PCR tested for proper integration of the construct using primer pairs oLC1093/oLC275 and oLC274/oLC1094. Transformants were additionally tested with primer pair oLC441/oLC1097 to verify presence of the *MAL2p-CDC37* allele and with primer pair oLC1093/oLC1097 to ensure no additional wild-type alleles of *CDC37* were present. The *SAP2* promoter was induced to drive expression of FLP recombinase to excise the NAT marker cassette.

**CaLC2276:** The *TAP-ARG4* cassette was amplified from pLC573 [9] with primer pair oLC2308/oLC2309 containing sequence homologous to *CNA1*, and transformed into CaLC239. ARG4+ prototrophic transformants were PCR tested for proper integration of the construct using primer pairs oLC1593/oLC2095 and oLC1594/oLC2096.

**CaLC2286:** The *TAP-ARG4* cassette was amplified from pLC573 [9] with primer pair oLC2308/oLC2309 containing sequence homologous to *CNA1*, and transformed into CaLC1779. ARG4+ prototrophic transformants were PCR tested for proper integration of the construct using primer pairs oLC1593/oLC2095 and oLC1594/oLC2096.

**CaLC2309:** The *TAP-ARG4* cassette was amplified from pLC573 [9] with primer pair oLC2308/oLC2309 containing sequence homologous to *CNA1*, and transformed into CaLC1966. ARG4+ prototrophic transformants were PCR tested for proper integration of the construct using primer pairs oLC1593/oLC2095 and oLC1594/oLC2096. The plasmid pLC583, containing the *SGT1-HA* tagging construct, was then digested with BssHII and transformed into CaLC1966 containing *CNA1-TAP*. NAT-resistant transformants were PCR tested for proper integration of the construct using primer pairs oLC300/oLC275 to verify the tagging construct integrated at the *tetO* locus, with primer pair and oLC274/oLC1632. The strain was additionally tested with primer pair oLC300/oLC1632 to verify the absence of additional untagged alleles of *SGT1*, and with oLC1741/oLC1742 to confirm presence of the deleted allele of *SGT1*.

**CaLC2310:** The *MAL2p-SGT1* construct was amplified from pLC625 with primer pair oLC1885/oLC1925 containing sequence homologous to *SGT1*, and transformed into CaLC1966. NAT-resistant transformants were PCR tested for proper integration of the construct using primer pairs oLC1741/oLC275 and oLC442/oLC1742 to verify proper integration of the *MAL2p-SGT1* cassette. The strain was additionally tested with primer pair oLC300/oLC1742 to verify the absence of the *tetO-SGT1* allele and oLC1839/oLC1840 to verify the absence of additional wild-type alleles of *SGT1*. The *TAP-ARG4* cassette was amplified from pLC573 [9] with primer pair oLC2308/oLC2309 containing sequence homologous to *CNA1*, and transformed into this *MAL2-SGT1/sgt1∆* strain. ARG4+ prototrophic transformants were PCR tested for proper integration of the construct using primer pairs oLC1593/oLC2095 and oLC1594/oLC2096. The plasmid pLC583, containing the *SGT1-HA* tagging construct, was then digested with BssHII and transformed into the *MAL2-SGT1/sgt1∆* strain containing *CNA1-TAP*. NAT-resistant transformants were PCR tested for proper integration of the construct using primer pairs oLC442/oLC275 to verify the tagging construct integrated at the *MAL2* locus, with primer pair and oLC274/oLC1632. The strain was additionally tested with primer pair oLC442/oLC1632 to verify the absence of additional untagged alleles of *SGT1*, and with oLC1741/oLC1742 to confirm presence of the deleted allele of *SGT1*.

**CaLC2278:** The *TAP-ARG4* cassette was amplified from pLC573 [9] with primer pair oLC2308/oLC2309 containing sequence homologous to *CNA1*, and transformed into CaLC432 [5]. ARG4+ prototrophic transformants were PCR tested for proper integration of the construct using primer pairs oLC1593/oLC2095 and oLC1594/oLC2096.

**Plasmid Construction**

**pLC624:** Sequence homologous to the start codon and beginning of the *SGT1* open reading frame was PCR amplified from SC5314 genomic DNA using primer pair oLC1924/oLC1925, digested with SacII and SacI, and ligated into pLC49 [3]. Integration was PCR tested using primer pair oLC274/oLC1925. The *tetO* promoter was excised from pLC330 [5] using NotI and SacII, and cloned into pLC49 containing sequence homologous to the start codon and beginning of the *SGT1* open reading frame. Integration was PCR tested using primer pair oLC274/oLC301. This plasmid can be amplified with primer pair oLC1885 (containing sequence homologous to *SGT1*) and oLC1925 in order to promoter replace *SGT1* with *tetO*.

**pLC625:** Sequence homologous to the start codon and beginning of the *SGT1* open reading frame was PCR amplified from SC5314 genomic DNA using primer pair oLC1924/oLC1925, digested with SacII and SacI, and ligated into pLC49 [3]. Integration was PCR tested using primer pair oLC274/oLC1925. The *MAL2* promoter was excised from pLC90 (pAU22 [10]) using NotI and SacII, and cloned into pLC49 containing sequence homologous to the start codon and beginning of the *SGT1* open reading frame. Integration was PCR tested using primer pair oLC274/oLC1925. This plasmid can be amplified with primer pair oLC1885 (containing sequence homologous to *SGT1*) and oLC1925 in order to promoter replace *SGT1* with *MAL2*.

**pLC583:** A portion of the C-terminus of the *SGT1* open reading frame was PCR amplified from SC5314 genomic DNA using primer pair oLC1622/oLC1623 (containing the *HA* tag), digested with KpnI and ApaI, and ligated into pLC49. Integration was PCR tested using primer pair oLC1622/oLC275. Sequence homologous to the downstream region of *SGT1* was PCR amplified from SC5314 genomic DNA using primer pair oLC1624/oLC1625, digested with SacII and SacI and ligated into pLC49 containing the C-terminus of *SGT1*. Integration was PCR tested using primer pair oLC274/oLC1625. The construct can be liberated using KpnI and SacI.

**pLC332:** The *MAL2* promoter was excised from plasmid pLC90 (pAU22 [10]) by digestion with NotI and SacII and ligated into pLC49.

**pLC505:** Sequence homologous to the upstream region of *CDC37* was PCR amplified from SC5314 genomic DNA using primer pair oLC1089/oLC1090, digested with ApaI, and ligated into pLC49. Integration was PCR tested using primer pair oLC1089/oLC275. Sequence homologous to the downstream region of *CDC37* was PCR amplified from SC5314 genomic DNA using primer pair oLC1091/oLC1092, digested with SacI and SacII, and ligated into pLC49 containing sequence homologous to the upstream region of *CDC37*. Integration was PCR tested using primer pair oLC274/oLC1092. This construct can be liberated using BssHII.

**pLC506:** Sequence homologous to the upstream region of *CDC37* was PCR amplified from SC5314 genomic DNA using primer pair oLC1089/oLC1090, digested with ApaI, and ligated into pLC332. Integration was PCR tested using primer pair oLC1089/oLC275. Sequence homologous the beginning of the *CDC37* ORF was PCR amplified from SC5314 genomic DNA using primer pair oLC1095/oLC1096, digested with SacI and SacII, and ligated into pLC332 containing sequence homologous to the upstream region of *CDC37*. Integration was PCR tested using primer pair oLC274/oLC1096. This construct can be liberated using BssHII.

**Growth Curves**

Yeast cells were grown overnight in YPD medium. Cells were diluted to OD_600_ of 0.0625 with or without doxycycline treatments as indicated in 96-well plates and grown at 30°C with continuous shaking, using the TECAN GENios. Optical density was measured at 595 nm every 15 minutes over 24 hours. Data was plotted and analyzed using Microsoft Excel.

**Cell Viability Plating**

Sgt1 levels were reduced by growth overnight in 20 µg/ml doxycycline, followed by subculture in fresh medium with 20 µg/ml doxycycline and growth until mid-log phase. Cultures were diluted and 100 µl of culture was plated on to YPD plates, and grown for 24 hours at 30˚C. Colony forming units (CFUs) were counted and plotted.

**Supplemental References**

1. Cowen LE (2009) Hsp90 orchestrates stress response signaling governing fungal drug resistance. PLoS Pathog 5: e1000471.

2. Morschhauser J, Michel S, Staib P (1999) Sequential gene disruption in *Candida albicans* by FLP-mediated site-specific recombination. Mol Microbiol 32: 547-556.

3. Shen J, Guo W, Kohler JR (2005) *CaNAT1*, a heterologous dominant selectable marker for transformation of *Candida albicans* and other pathogenic *Candida* species. Infect Immun 73: 1239-1242.

4. Noble SM, Johnson AD (2005) Strains and strategies for large-scale gene deletion studies of the diploid human fungal pathogen *Candida albicans*. Eukaryot Cell 4: 298-309.

5. Shapiro RS, Uppuluri P, Zaas AK, Collins C, Senn H, et al. (2009) Hsp90 orchestrates temperature-dependent *Candida albicans* morphogenesis via Ras1-PKA signaling. Curr Biol 19: 621-629.

6. Fang HM, Wang Y (2006) RA domain-mediated interaction of Cdc35 with Ras1 is essential for increasing cellular cAMP level for *Candida albicans* hyphal development. Mol Microbiol 61: 484-496.

7. Robbins N, Collins C, Morhayim J, Cowen LE (2009) Metabolic control of antifungal drug resistance. Fungal Genet Biol 47: 81-93.

8. Wiederhold NP, Grabinski JL, Garcia-Effron G, Perlin DS, Lee SA (2008) Pyrosequencing to detect mutations in *FKS1* that confer reduced echinocandin susceptibility in *Candida albicans*. Antimicrob Agents Chemother 52: 4145-4148.

9. Lavoie H, Sellam A, Askew C, Nantel A, Whiteway M (2008) A toolbox for epitope-tagging and genome-wide location analysis in *Candida albicans*. BMC Genomics 9: 578.

10. Uhl MA, Johnson AD (2001) Development of Streptococcus thermophilus *lacZ* as a reporter gene for *Candida albicans*. Microbiology 147: 1189-1195.

11. Cowen LE, Singh SD, Kohler JR, Collins C, Zaas AK, et al. (2009) Harnessing Hsp90 function as a powerful, broadly effective therapeutic strategy for fungal infectious disease. Proc Natl Acad Sci USA 106: 2818-2823.
